# Antimicrobial Resistance and Genomic Characterization of *Salmonella* Isolated from Pigeons in China

**DOI:** 10.1155/2024/3315678

**Published:** 2024-05-06

**Authors:** Yuhua Zhang, Zheng Lu, Haoyu Zhao, Shuangyu Li, Hong Zhuang, Juan Wang, Ruichao Li, Weibo Zheng, Hongwei Zhu, Peng Xie, Yibin Hu, Caiyuan Zhou, Qian Mao, Leilei Sun, Shanshan Li, Wenhui Wang, Fang Wang, Wei Pan, Chengbao Wang

**Affiliations:** ^1^College of Veterinary Medicine, Northwest A&F University, Yangling 712100, Shaanxi, China; ^2^College of Veterinary Medicine, Yangzhou University, Yangzhou 225009, Jiangsu, China; ^3^School of Life Sciences, Ludong University, Yantai 264025, Shandong, China; ^4^Jiangsu Key Laboratory of Immunity and Metabolism, Jiangsu International Laboratory of Immunity and Metabolism, Department of Pathogen Biology and Immunology, Xuzhou Medical University, Xuzhou 221004, Jiangsu, China

## Abstract

Salmonellosis is one of the important bacterial infectious diseases affecting the health of pigeons. Heretofore, the epidemiological characteristics of *Salmonella* in pigeon populations in China remain largely unclear. The present study investigated the antimicrobial resistance and genomic characteristics of *Salmonella* isolates in pigeons in different regions of China from 2022 to 2023. Thirty-two *Salmonella* isolates were collected and subjected to 24 different antimicrobial agents, representing nine categories. The results showed that these isolates were highly resistant to cefazolin (100%), gentamicin (100%), tobramycin (100%), and amikacin (100%). Three or more types of antimicrobial resistance were present in 90.62% of the isolates, indicating multidrug resistance. Furthermore, using whole genome sequencing technology, we analyzed the profiles of serotypes, multilocus sequence typing, virulence genes, antimicrobial resistance genes, and plasmid replicons and constructed phylogenetic genomics to determine the epidemiological correlation among these isolates. All strains belonged to *Salmonella* Typhimurium var. Copenhagen and exhibited five antimicrobial resistance genes and more than 150 *Salmonella* virulence genes. Moreover, each isolate contained both the IncFIB(S) and IncFII(S) plasmids. In addition, phylogenetic analysis showed that all isolates were very close to each other, and isolates from the same region clustered in the same branch. Overall, our findings provide the first evidence for the epidemiological characteristics of *Salmonella* in pigeons of China, highlighting the importance of preventing salmonellosis in pigeons.

## 1. Introduction

*Salmonella* is one of the main pathogens leading to the global outbreak of foodborne illness, which poses a serious threat to human health worldwide [1]. In China, 70%–80% of bacteria-related foodborne illnesses are caused by *Salmonella* infection [2]. Recent surveys of the disease burden in multiple Chinese provinces have shown that the annual incidence of *Salmonella* is roughly 245/100,000 [3]. Moreover, more than 2,610 *Salmonella* serotypes have been identified, many of which are pathogenic to both humans and animals [4, 5]. Notably, *Salmonella*-infected animals, including mice, wild animals, fowl, and domestic livestock, are important sources of *Salmonella* contamination in humans. Thus, it is of great importance to monitor the epidemiological profiles of *Salmonella* infections in animals.

Due to a close contact with humans, pigeons present one important source where humans acquire *Salmonella* infections. For example, *Salmonella* in meat pigeons can infect humans *via* the food chain, while racing pigeons can transmit the pathogen through direct contact and feces [6, 7]. Salmonellosis can spread not only horizontally but also vertically. For instance, *Salmonella* can infect breeding pigeons and then spread to the eggs through vertical transmission, resulting in egg necrosis and a decrease in the hatching rate. It can also infect squabs *via* lactation, leading to infection or even death. Moreover, any age of pigeons can be infected with *Salmonella* [8], causing a large number of deaths [9]. In addition, *Salmonella* Typhimurium (*S*. Typhimurium) is one of the most important serotypes of *Salmonella* Enteritidis, which can infect both humans and a range of animal hosts due to its excellent survival ability under different dietary conditions [10].

In China, the industry for pigeon racing has grown, and meat pigeon consumption has increased in recent years. Accumulating evidence has shown the infection of *Salmonella* in pigeons, with *S*. Typhimurium infections as the majority species. For example, *S*. Typhimurium is reported to be the dominant serotype of pigeon *Salmonella* isolates in Guangdong and Shanghai, China, and Poland [11–13]. Infected pigeons often show single or multiple serious clinical symptoms, including diarrhea, weight loss, limps, head and neck skews, and other neurological symptoms [13, 14]. Furthermore, the misuse and abuse of antibiotics to treat salmonellosis has led to the emergence of multidrug resistance (MDR) bacteria [15, 16]. Therefore, a comprehensive epidemiological study and MDR profiles are needed to clarify the potential danger of *Salmonella* infection to pigeons and human health in China. Whole genome sequencing (WGS) can provide accurate information on population dynamics, genome epidemiology, and bacterial genomic characteristics [17]. A previous study has used WGS to analyze the serotype, antimicrobial resistance, multilocus sequence typing (MLST), virulence genes, and plasmid replicons of *Salmonella* strains in the United States [18]. However, the WGS profiles of *Salmonella* isolates in Chinese pigeon populations are still unknown.

In the present study, we investigated the prevalence of *Salmonella* in racing pigeon clubs and meat pigeon farms across various regions of China. A total of 32 *Salmonella* strains were isolated from pigeons. Phenotypic resistance results showed that 90.62% of the isolates were MDR. Furthermore, WGS in conjunction with bioinformatics analysis was used to investigate the genomic characteristics of *Salmonella* isolates. We found that all isolates were *Salmonella* Typhimurium var. Copenhagen, and the resistance genes, virulence genes, and plasmid types carried by them were also very similar. Overall, these results contribute to a better understanding of the epidemiology and transmission mechanisms of *Salmonella* in pigeon populations in China.

## 2. Materials and Methods

### 2.1. Ethics Statement

This study was carried out in accordance with standard procedures without any operations on living animals. Dead pigeons with clinical symptoms of *Salmonella* infection were used as a sample source for this study.

### 2.2. Sample Collection

**Figure 1:**
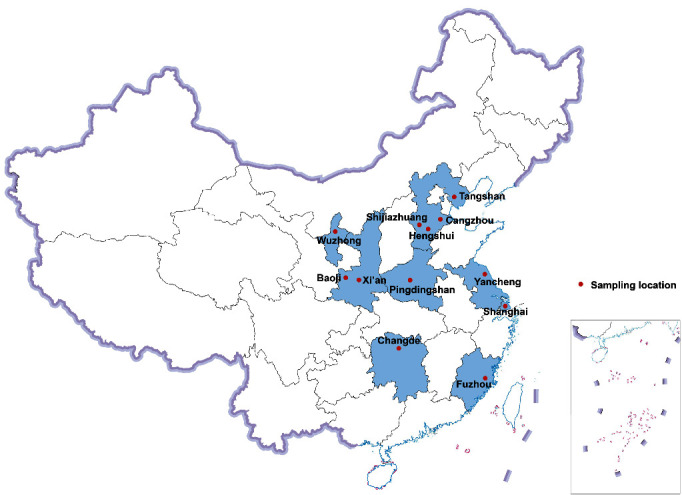
Distribution of sampling locations in eight provinces and regions of China.

Between June 2022 and April 2023, 215 samples were gathered for this investigation, comprising 92 samples of racing pigeons and 123 samples of meat pigeons. This study was not a random sampling, but a sample of sick pigeons with clear clinical symptoms was selected through clinical diagnosis. These collected samples showed weight loss, head and neck deviation, arthritis, diarrhea, and other single or comprehensive symptoms. Details of the sample collection are shown in Table 1. These samples were collected from four cities in Hebei Province, two cities in Shaanxi Province, Henan Province, Hunan Province, Jiangsu Province, Fujian Province, Ningxia Hui Autonomous Region, and Shanghai (Figure 1).

### 2.3. Isolation and Identification of *Salmonella* Isolates

The procedure for *Salmonella* isolation is depicted as follows: Following aseptic necropsy, tissue samples from the liver, heart, kidney, lung, and intestine were collected for *Salmonella* isolation (Figure S1). To increase the number of bacteria in tissue samples, the proper amounts of the tissue samples were inoculated in a selenite cystine solution (Haibo biology, HB4085, China) and cultured for 18 hr at 37°C. Then, the red precipitate's culture solution was smeared on agar medium containing xylose–lysine–deoxycholic (XLD) (Haibo biology, HB4105, China) acid and cultured at 37°C for 24 hr to identify potential *Salmonella* colonies. Finally, characteristic black center colonies or colorless translucent colonies were chosen to identify *Salmonella* isolates using 16S rDNA PCR (Table S1 and Figure S2).

### 2.4. Antimicrobial Susceptibility Testing

According to the Clinical Laboratory Standard Institute (CLSI) guidelines, antimicrobial susceptibility profiles were assessed by broth microdilution using the Sensititre™ automated antimicrobial susceptibility system (Thermo Fisher Scientific, United Kingdom) and the Gram-negative GN4F plate. The 24 antimicrobial agents tested were as follows: penicillins (ampicillin: AMP, 8–16 *μ*g/mL; piperacillin: PIP, 16–64 *μ*g/mL; ticarcillin/clavulanic acid: TCC, 8/2−64/2 *μ*g/mL; ampicillin/sulbactam: SAM, 4/2−16/8 *μ*g/mL; piperacillin/tazobactam: TZP, 8/4−128/4 *μ*g/mL), carbapenems (meropenem: MEM, 0.5–8 *μ*g/mL; ertapenem: ETP, 0.25–8 *μ*g/mL; imipenem: IPM, 0.5–8 *μ*g/mL; doripenem: DOR, 0.5–4 *μ*g/mL), cephalosporins (cefazolin: CZO, 1–16 *μ*g/mL; cefepime: FEP, 4–32 *μ*g/mL; ceftazidime: CAZ, 1–16 *μ*g/mL; ceftriaxone: CRO, 0.5–32 *μ*g/mL), monobactams (aztreonam: ATM, 1–16 *μ*g/mL), aminoglycosides (gentamicin: GEN, 2–8 *μ*g/mL; amikacin: AMK, 8–32 *μ*g/mL; tobramycin: TOB 2–8 *μ*g/mL), tetracyclines (tetracycline: TET, 4−8 *μ*g/mL; minocycline: MIN, 1–8 *μ*g/mL; tigecycline: TGC, 1–8 *μ*g/mL), quinolones (ciprofloxacin: CIP, 0.5–2 *μ*g/mL; levofloxacin: LVX, 1−8 *μ*g/mL), folate pathway inhibitors (trimethoprim/sulfamethoxazole: TMS, 2/38−4/76 *μ*g/mL), and nitrofuran (nitrofurantoin: NIT, 32–64 *μ*g/mL). The minimum inhibitory concentration (MIC) for each antibiotic was interpreted using CLSI standards and NARMS breakpoints, and the MIC values were categorized as susceptible (S), intermediate (I), or resistant (R). In order to facilitate the analysis of the results, the sensitive intermediate-resistant strains were identified as having antimicrobial resistance, and the strains with three or more antimicrobial resistances were identified as having MDR. The quality control strain utilized was *Escherichia coli* ATCC 25922.

### 2.5. Genomic DNA Extraction and Whole Genome Sequencing

The isolated strains were shaken overnight at 37°C in Luria–Bertani (LB) (Haibo biology, HB0128, China) liquid medium, and then centrifuged to obtain a bacterial precipitate. Bacterial genomic DNA was extracted using a commercial TIANmp Bacterial DNA kit (Tiangen Biotech, China) according to the manufacturer's instructions. After passing the DNA sample quality test, a sequencing library was generated using the Rapid Plus DNA Lib Prep Kit for Illumina (Cat. No. RK20208). WGS of all isolates was performed at Novogene Bioinformatics Technology Co., Ltd. (Tianjin, China) using Illumina platforms and the PE150 strategy.

### 2.6. Bioinformatic Analyses

After performing quality checks on the raw reads with Fastp (v0.23.1) [19], the reads were assembled with SPAdes 4.0.1 [20] using the “careful” command option. After genome assembly, SeqSero1.2 (https://cge.food.dtu.dk/services/SeqSero/) [21] and MLST2.0 (https://cge.food.dtu.dk/services/MLST/) were used to predict the serotype and sequence type of the isolated strains on the data platform of the Genome Epidemiology Center [22]. PlasmidFinder 2.1 (https://cge.food.dtu.dk/services/PlasmidFinder/) was additionally employed to find the plasmid type with a minimum coverage of 60% and a nucleotide identity of 95% [23]. In addition, the virulence factor database (VFDB) (http://www.mgc.ac.cn/cgi-bin/VFs/v5/main.cgi?func=VFanalyzer) manages the virulence factors of bacterial pathogens, and we conducted virulence gene prediction for 32 *Salmonella* isolates here [24]. In order to conduct a comprehensive analysis of resistance genes as much as possible, the resistance genes were detected using ResFinder 4.4.1 (https://cge.cbs.dtu.dk/services/ResFinder/) with settings of threshold of 95% and minimum length of 60%. Moreover, using homology and single nucleotide polymorphism (SNP) model prediction of resistance groups on the comprehensive antibiotic resistance database platform, the Resistance Gene Identifier (https://card.mcmaster.ca/analyze/rgi) software was used to identify the AMR gene [25]. Additionally, an evolutionary tree based on the SNPs of the core genomes was built using the maximum likelihood method (Roary and FastTree). In this study, the phylogenetic tree, corresponding serotype, ST (sequence type), province, antimicrobial resistance genes, and insertion sequences were visualized using the Interactive Tree of Life (https://itol.embl.de) web server.

## 3. Results

### 3.1. Prevalence and Serotypes of *Salmonella* in Pigeons

**Figure 2:**
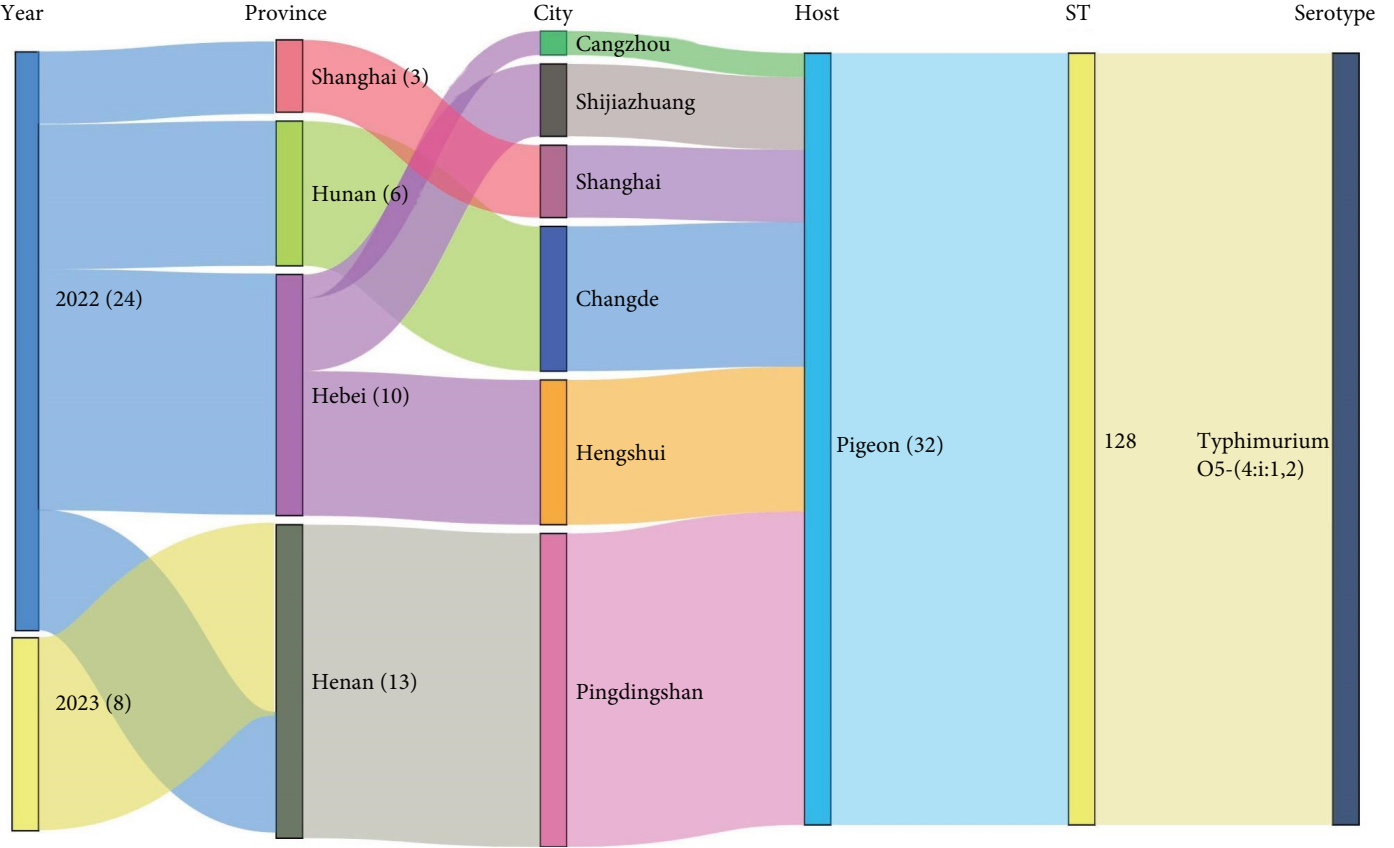
Distribution of *Salmonella* isolates studied according to sampling year, province, host, sequence type, and serotype.

Among all meat pigeon samples (*n* = 123), 32 *Salmonella* isolates were identified, mainly distributed in Henan, Hebei, Hunan, and Shanghai in China. Of the 96 racing pigeon samples collected from five provinces, no *Salmonella* isolate was found. In the entire pigeon population, the prevalence of *Salmonella* was 14.9%, while the prevalence in meat pigeons was 26%. Interestingly, every isolate shared the same serotype and MLST pattern and had similar copies of the seven housekeeping genes: *aroC*, *hemD*, *purE*, *thrA*, *dnaN*, *sucA*, and *hisD*. All isolates were *Salmonella* Typhimurium var. Copenhagen ST128 (*n* = 32) (Figure 2).

### 3.2. Phenotypic Antimicrobial Resistance

**Figure 3:**
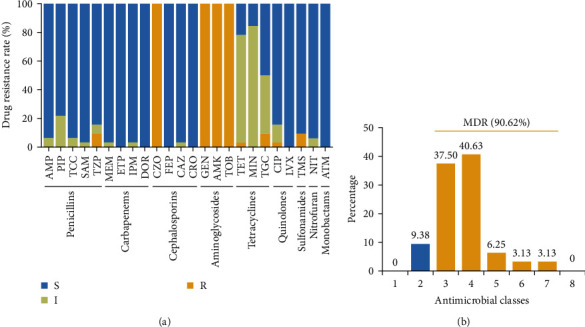
Phenotypic antimicrobial susceptibility (a) and distribution of multiple resistance among the studied *Salmonella* isolates (b). Abbreviations: AMP, ampicillin; PIP, piperacillin; TCC, ticarcillin/clavulanic acid; SAM, ampicillin/sulbactam; TZP, piperacillin/tazobactam; MEM, meropenem; ETP, ertapenem; IPM, imipenem; DOR, doripenem; CZO, cefazolin; FEP, cefepime; CAZ, ceftazidime; CRO, ceftriaxone; GEN, gentamicin; AMK, amikacin; TOB, tobramycin; TET, tetracycline; MIN, minocycline; TGC, tigecycline; CIP, ciprofloxacin; LVX, levofloxacin; TMS, trimethoprim/sulfamethoxazole; NIT, nitrofurantoin; and ATM, aztreonam.

All *Salmonella* isolates were subjected to testing for 24 different antimicrobial drugs in nine different categories, and the results are shown in Table S2 and Figure S3. In order to facilitate the statistical results, we classified intermediate drug-resistant strains as drug-resistant results. We found high resistance of isolates to gentamicin, tobramycin, cefazolin, and amikacin (100%; 32/32), followed by minocycline (84.375%; 27/32), tetracycline (78.125%; 25/32), and tigecycline (50%; 16/32). Moreover, all isolates were susceptible to cefepime, ceftriaxone, doripenem, ertapenem, aztreonam, and levofloxacin (Figure 3(a)). In addition, 90.62% of the strains were resistant to at least three types of antibiotics, indicating MDR (Figure 3(b)).

### 3.3. Genotypic Antimicrobial Resistance

**Figure 4:**
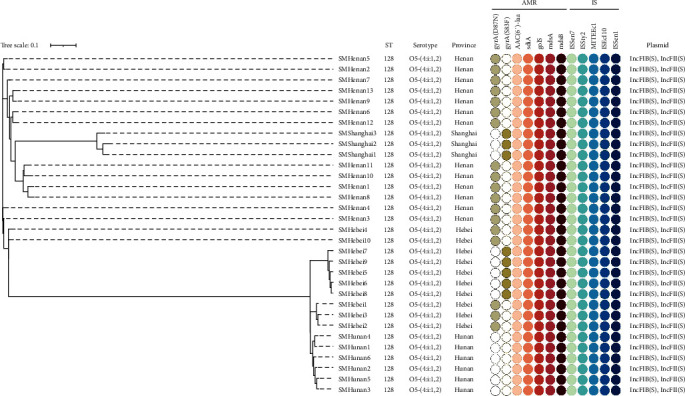
Phylogenetic tree and statistical combination of 32 strains of *Salmonella* from pigeons in China based on SNP analysis.

Analyzing the antimicrobial resistance genes, 32 *Salmonella* isolates were identified to carry five identical antimicrobial resistance genes. All isolates contained aminoglycoside acetyltransferase *aac* (*6′*)-*Iaa* resistance genes, efflux mechanism genes *mdsA* and *mdsB*, genes encoding gold resistance *golS*, and biofilm-related genes *sdiA* (Figure 4). Moreover, genomic mutations conferring quinolone resistance were detected in 26 (81.25%) isolates, and two single mutations of the *gyrA* gene were observed: *gyrA* (D87N) (*n* = 18) and *gyrA* (S83F) (*n* = 8) (Figure 4).

### 3.4. Plasmid Replicons

Using the PlasmidFinder tool, we detected the plasmids carried by *Salmonella* strains. These isolates carried the same plasmid replicon, and a total of two plasmids were detected in 32 isolates. The plasmids of isolates were IncFII(S) and IncFIB(S) (Figure 4). Interestingly, the IncFll(S) and IncFIB(S) plasmids were carried by different isolates from pigeons from different provinces, indicating that these plasmids have widespread distribution across different geographical regions.

### 3.5. *Salmonella* Virulence Genes

**Figure 5:**
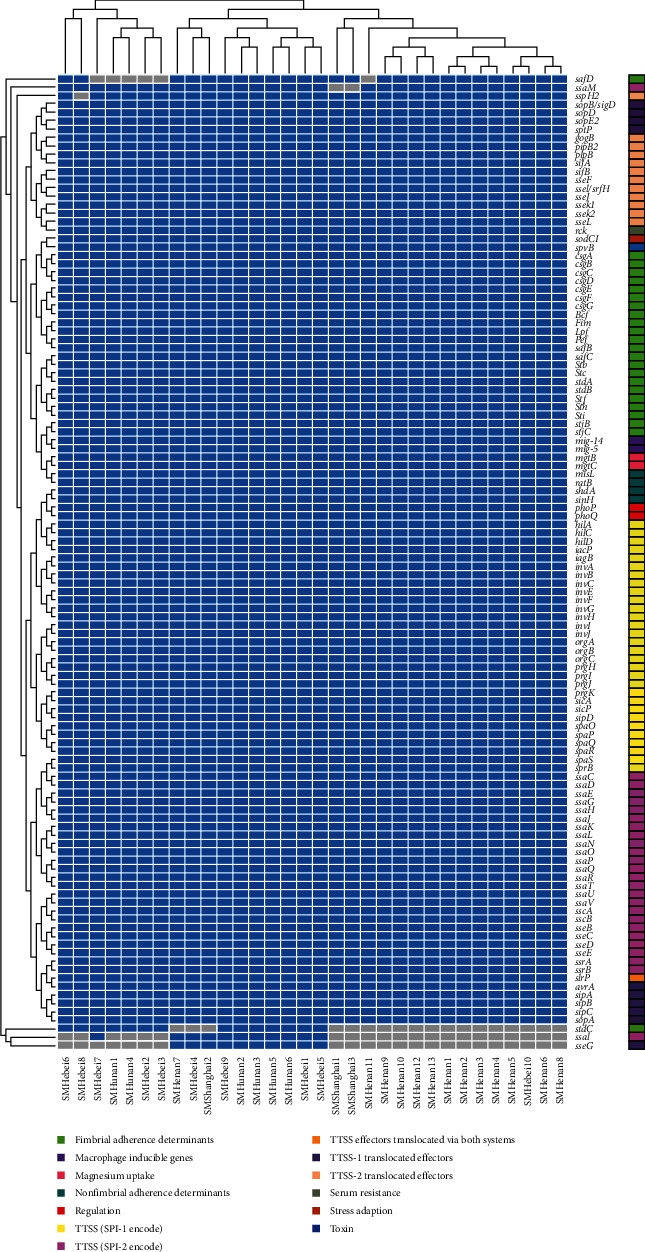
Heatmap of tree hierarchical clustering of virulence genes and isolated strains. This figure shows the prediction of the virulence gene profile of the studied isolates. The *x*-axis shows the isolate ID numbers, and the *y*-axis shows the identified selected virulence genes. Blue cells indicate the presence of a gene; gray cells indicate the absence of a gene.

In order to investigate the virulence gene profile of *Salmonella* isolates, we analyzed the genome with the complete VFDB dataset. A total of 156 genes associated with the virulence and pathogenicity mechanisms of *Salmonella* were found in the 32 *Salmonella* strains. The number of virulence genes in each isolate ranged from 151 to 156, and there was little difference in virulence genes among isolates (Figure 5). Moreover, the distribution of *S*. Typhimurium virulence genes isolated from various geographical locations was nearly the same, with only one to six virulence gene differences. There were 1–6 virulence gene differences among different isolates, including *safD*, *stdC*, *ssaI*, *ssaM*, *sseG*, and *sspH2*. All *Salmonella* isolates had plasmids harboring the *spvB* gene, the stress adaptation gene *sodCI*, the enterotoxin gene *stn*, and the serum resistance gene *rck*, all of which are critical components of the virulence system of *Salmonella*. In addition, the typical virulence genes from *Salmonella* pathogenic islands 1 and 2 (SPI-1 and SPI-2) were present in all *Salmonella* strains analyzed.

## 4. Discussion

As an important zoonotic pathogen, *Salmonella* is seriously harmful to humans and animals, causing huge economic losses. The pigeon breeding industry is the fourth major poultry breeding industry in China [26]. However, so far, the pigeon industry has not yet established a strict biosafety management system, and there are also defects in disease prevention and immunization procedures. Therefore, once a *Salmonella* infection occurs, it will cause serious damage to pigeons. The present study investigated the epidemiological, antimicrobial resistance, and genomic characteristics of *Salmonella* isolated from pigeons in various parts of China using WGS data in conjunction with accurate bioinformatics techniques, which provides a scientific basis for the spread of *Salmonella* in pigeons in China.

In this study, 32 strains of *Salmonella* from pigeons were all *Salmonella* Typhimurium var. Copenhagen. MLST analysis showed that all strains belong to ST128. Previous studies have reported that *S*. Typhimurium is the most common serotype of *Salmonella* in animals [27]. Moreover, the Copenhagen variant is the most prevalent variant of *S*. Typhimurium in pigeons [28]. It is reported that the sequence type of *S*. Typhimurium isolated from pigeons from China and Poland was also ST128. These results, including ours, indicate that the serotype and sequence type of *Salmonella* isolates may be a stable lineage in the pigeon population. Furthermore, we showed that the prevalence of *S*. Typhimurium in the overall pigeon population was 14.9%, and in meat pigeons, it was 26%. In line with our finding, two previous studies reported 26.1% and 21% prevalence of *S*. Typhimurium in the meat pigeon population in the markets of Shanghai and Beijing, respectively [12, 29]. The prevalence of salmonellosis in pigeons in Italy, Egypt, and Poland was reported to be 0.9%, 13.3%, and 5.5%, respectively [13, 30, 31]. The prevalence of salmonellosis in pigeons varies in different countries, which may be related to different sampling methods. But it is worth noting that the situation of *Salmonella* infection in meat pigeon populations in China may be more severe, which needs extensive investigation in pigeon populations.

Antibiotics are the most commonly therapy for the treatment of bacterial diseases, including pigeon salmonellosis. In the field of veterinary medicine, the widespread and sustained use of antimicrobial agents has led to the development of AMR in animals and livestock [32]. The present study evaluated the phenotypic and genotypic AMR profiles of 32 *Salmonella* strains in pigeons. In order to identify accurate epidemiological information about the antimicrobial susceptibility of *Salmonella*, all isolates were tested for 24 different antimicrobial agents. In the present study, the phenotypic resistance results of amikacin, gentamicin, and tobramycin were consistent with the aminoglycoside resistance gene *aac* (*6′*)-*Iaa*, and there was no significant correlation between the resistance of other types of antibiotics and the resistance genes carried. Antimicrobial resistance genes do not necessarily mean phenotypic resistance and vice versa. The occurrence of antimicrobial resistance is not only determined by the presence of antimicrobial resistance genes but also affected by various factors, such as enzyme activation, target modification or protection, and biofilm formation [33]. We found that all strains exhibited a common resistance to gentamicin, amikacin, tobramycin (aminoglycosides), and cefazolin (first-generation cephalosporins), although they come from different regions. The result was also observed in the antimicrobial susceptibility test of *Salmonella* isolated from humans [34]. We also found that some strains were resistant to tigecycline, but no plasmid-mediated tigecycline resistance genes *tet* (X3) and *tet* (X4) were found. It is speculated that the causes of antimicrobial resistance are related to other tigecycline resistance mechanisms, such as overexpression of efflux pumps, mutation of cell membrane porin, and ribosome protection mechanisms [35]. Notably, we showed that 90.62% of *Salmonella* isolates in pigeons were MDR and could resist at least three types of antimicrobial agents. Considering the limited antibiotics available to pigeons at present, infection with MDR strains may lead to ineffective treatment. This reminds us to continuously monitor antimicrobial resistance in pigeon populations and use antibiotics reasonably.

The antimicrobial resistance genes predicted by the two data platforms are slightly different. Therefore, we combined the antimicrobial resistance genes and chromosome mutation results with a threshold consistency higher than 95% in the two data platforms for statistics. Antimicrobial resistance can be more accurately predicted when the two methods are combined. We detected five antimicrobial resistance genes in *Salmonella* isolates from pigeons. Among them, the most common resistance gene is *aac* (*6′*)-*Iaa*, which encodes aminoglycoside drugs. Moreover, phenotypic resistance studies confirmed that all strains were resistant to aminoglycosides (gentamicin, tobramycin, and amikacin). Consistent with a previous study [36], all isolates were positive for the gene *aac* (*6′*)-*Iaa* encoding aminoglycoside resistance. In addition, we found that the efflux pump of the *mdsABC* complex encodes the antimicrobial resistance genes *mdsA* and *mdsB*. *MdsABC* is a unique multidrug transport factor for *Salmonella*. *MdsA* and *mdsB* can promote resistance to various antibacterial drugs, including phenicol antibiotic, cephalomycin, monobactam, carbapenem, and cephalosporin. This indicates that its presence can lead to resistance to multiple antibiotics [37–40]. Furthermore, partial regulatory systems contribute to bacterial resistance to heavy metals. In *Salmonella*, the response to gold ions is mediated by a specific metal regulatory factor *golS*, which controls the expression of the RND (resistance–nodulation–division) efflux pump gesABC. The CpxR/CpxA system (cell-envelope stress-responding system) promotes the gold resistance of *Salmonella* by controlling gols-dependent gesABC transcription, enabling microorganisms to resist contaminated environments [41–43]. Notably, the *sdiA* gene exists in all isolated strains. To our best knowledge, there is no study to clarify the role of the *sdiA* gene in antimicrobial resistance. The *sdiA* gene of *Salmonella* regulates the *rck* gene, which mediates the adhesion and invasion of *Salmonella* to epithelial cells and the body's resistance to complement [44, 45]. In addition, we observed mutations caused by amino acid substitutions in *gyrA*, from serine to phenylalanine at codon 83 (S83F) and from aspartic acid to asparagine at codon 87 (D87N). Single point mutations in the quinolone resistance-determining region of *gyrA* reduce the sensitivity of fluoroquinolones (e.g., ciprofloxacin). However, antimicrobial resistance may require two or more mutations in the quinolone-determining region of *gyrA*, *gyrB*, *parC*, and *parE* [46–48].

Plasmids, transferred between different bacterial species and clones, can carry antimicrobial resistance or virulence genes. Obtaining plasmids carrying antimicrobial resistance or virulence genes is transmitted to each other between bacteria of different and geographical origins, which may alter the prevalence of virulence or multidrug-resistant bacterial cloning [49, 50]. The present study found IncFIB(S) and IncFII(S) plasmids in all isolates. IncFIB(S) and IncFII(S) plasmids are *Salmonella* virulence plasmids that may carry virulence-related genes *spv*, *rck* (resistance to complement killing), and *pef* (plasmid-encoded fimbriae) [51]. However, antimicrobial genes were not found on the contigs carrying the IncFIB(S) and IncFII(S) sequence replicons. This may have been due to the absence of these genes on virulence plasmids [23].

Virulence genes are closely related to the ability of *Salmonella* to invade host cells and spread *in vivo* [52]. In this study, we found 156 genes related to *Salmonella* virulence and pathogenic mechanisms in the genomes of 32 *Salmonella* isolates in pigeons. All isolates carried the typical virulence factors of *Salmonella* pathogenicity islands 1 and 2, indicating the strong pathogenicity of these isolates. There were 1–6 virulence gene differences among different *Salmonella* isolates; however, the serum resistance gene *rck*, the stress adaptation gene *sodCI*, the enterotoxin gene *stn*, and plasmid-carrying *spvB* gene were detected in each *Salmonella* isolate. It is reported that *rck* functions as a “gene toxin” that affects the DNA integrity of epithelial cells, in addition to its roles as a “cyclomodulin” that affects the cell cycle machinery and as an invasion factor [53]. The *sodCI* gene decreases external oxidative damage to host cells by encoding phagocytic superoxide dismutase [54]. The enterotoxin *stn* gene has a significant role in the pathogenicity of *S*. Typhimurium infection, which can maintain the composition and integrity of the bacterial cell membrane by regulating the localization of the OmpA membrane [55]. The *spvB* virulence gene of *Salmonella* acts as an intracellular ADP-ribosylation toxin, which can rearrange the host cytoskeleton to increase *Salmonella* invasion and promote systemic transmission [56, 57].

Overall, the *Salmonella* strains prevalent in Chinese pigeon populations were *Salmonella* Typhimurium var. Copenhagen, although these isolates were isolated in different regions and at different times. The analysis of the whole genome sequence characteristics showed that these strains had highly similar antimicrobial resistance genes and virulence genes along with the same sequence type, serotype and plasmid replicons. In addition, the genetic relationship between isolates from different regions is very close, indicating that there may be a pandemic clonal transmission of pigeon salmonellosis in China. Studies have reported that the *Salmonella* Typhimurium var. Copenhagen can be transmitted to humans and other animals [7, 9, 58]. This suggests that *Salmonella* originating from pigeons may pose a threat to human health. Therefore, the prevention and control of salmonellosis should be strengthened in the pigeon industry.

## 5. Conclusions

Using WGS combined with bioinformatics analysis methods, this study provides accurate information on the epidemiology of *Salmonella* in pigeon flocks from different regions of China. Even if there are several limitations to the present study, such as a possible insufficient sample size, the results nevertheless carry significant reference value. We found that *Salmonella* typhimurium Copenhagen variants are the common serotype prevalent in pigeons in different regions of China. Notably, 90.62% of the isolates showed multidrug resistant, which deserves more investigations. Meanwhile, there are large number of virulence genes carried by the isolated strains, indicating a strong pathogenicity and virulence and consequently, a serious harm to pigeon industry. Overall, the present study reveals the urgency of strengthening the research and monitoring of *Salmonella* epidemiology in pigeons.

## Figures and Tables

**Table 1:** The source and prevalence statistics of *Salmonella* collected samples in this study.

Province	City	Breed	Sample	*Salmonella* isolates	Prevalence rate (%)
Hunan	Changde	Meat pigeon	30	6	20.0
Hebei	Shijiazhuang	Meat pigeon	6	3	50.0
Hebei	Hengshui	Meat pigeon	17	6	35.3
Hebei	Cangzhou	Meat pigeon	8	1	12.5
Henan	Pingdingshan	Meat pigeon	37	13	35.1
Shanghai	Shanghai	Meat pigeon	25	3	12.0
Hebei	Tangshan	Racing pigeon	5	0	0
Shaanxi	Xi'an	Racing pigeon	25	0	0
Shaanxi	Baoji	Racing pigeon	8	0	0
Jiangsu	Yancheng	Racing pigeon	30	0	0
Fujian	Fuzhou	Racing pigeon	15	0	0
Ningxia	Wuzhong	Racing pigeon	9	0	0

## Data Availability

The data used to support the findings of this study are included within the article.

## Supplementary Materials

Supplementary Materials Figure S1: part of the samples in this study were dissected for clinical symptoms. Figure S2: the results of PCR amplification of 16S rDNA gene of some *Salmonella* isolates. Figure S3: phenotypic antimicrobial resistance heat map of *Salmonella* isolates studied. Table S1: PCR primers used in this study. Table S2: summary of detailed data involved in this study.
