# Supplementary material for: Antimicrobial Resistance and Genomic Characterization of Salmonella Isolated from Pigeons in China
Source: Transbound Emerg Dis. 2024 May 6;2024:3315678. doi: 10.1155/2024/3315678 (PMC12017069; doi:10.1155/2024/3315678)
Supplement: Supplementary Materials — Figure S1: part of the samples in this study were dissected for clinical symptoms. Figure S2: the results of PCR amplification of 16S rDNA gene of some Salmonella isolates. Figure S3: phenotypic antimicrobial resistance heat map of Salmonella isolates studied. Table S1: PCR primers used in this study. Table S2: summary of detailed data involved in this study. [file 3315678.f1.docx]

Transboundary And Emerging Diseases

# Antimicrobial resistance and genomic characterization of *Salmonella* isolated from Pigeons in China

Yuhua Zhang^1#^, Zheng Lu ^1#^, Haoyu Zhao^1#^, Shuangyu Li^1#^, Hong Zhuang^1#^, Juan Wang^1^, Ruichao Li^3^, Weibo Zheng^4^, Hongwei Zhu^4^, Peng Xie^1^, Yibin Hu^1^, Caiyuan Zhou^1^, Qian Mao^1^, Leilei Sun^1^, Shanshan Li^1^, Wenhui Wang^1^, Fang Wang^1^, Wei Pan^2^*, Chengbao Wang^1^*

^1^ College of Veterinary Medicine, Northwest A&F University, Yangling, 712100, Shaanxi, China

^2^ Jiangsu Key Laboratory of Immunity and Metabolism, Jiangsu International Laboratory of

Immunity and Metabolism, Department of Pathogen Biology and Immunology, Xuzhou Medical University, Xuzhou 221004, Jiangsu, China

^3^College of Veterinary Medicine, Yangzhou University, Yangzhou 225009, Jiangsu, China

^4^School of Life Sciences, Ludong University, Yantai, 264025, Shandong, China

Email addresses:

Yuhua Zhang: zhangyuhuayhh@163.com

Zheng Lu: lz_mixueer@163.com

Haoyu Zhao: zhaohaoyu982022@163.com

Shuangyu Li: lishuangyu11@163.com

Hong Zhuang: zhuanghong1984@sina.com

Juan Wang: juan.wang1234@hotmail.com

Ruichao Li: rchl88@yzu.edu.cn

Weibo Zheng: wbzheng@foxmail.com

Hongwei Zhu: hwzhu@ldu.edu.cn

Peng Xie: [xie.peng@drbata.net](mailto:xie.peng@drbata.net)

Yibin Hu: [ybhulove@163.com](mailto:ybhulove@163.com)

Caiyuan Zhou: [caiyuanzhou@sina.com](mailto:caiyuanzhou@sina.com)

Qian Mao:maoqian@stu.scau.edu.cn

Leilei Sun: 337499951@qq.com

Shanshan Li: 17792584320@163.com

Wenhui Wang: 18093851013@163.com

Fang Wang: q411124@163.com

#These authors contributed equally to this study

*Correspondence authors

Correspondence should be addressed to Chengbao Wang, Northwest A&F University, E-mail: [wangchengbao@nwsuaf.edu.cn](mailto:wangchengbao@nwsuaf.edu.cn) and Wei Pan, Xuzhou Medical University, E-mail: panwei525@126.com

**Figure S1. Part of the samples in this study were dissected for clinical symptoms.**


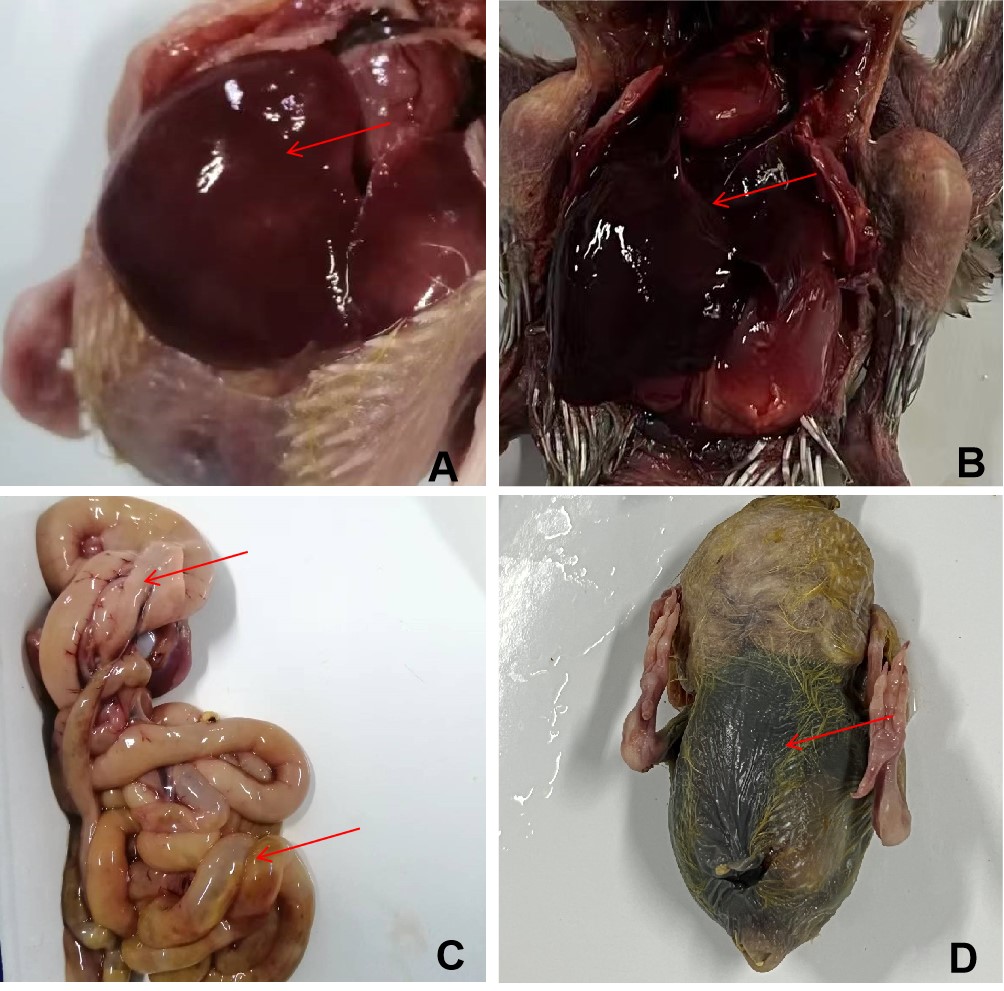


A. The liver features marked enlargement and brittle texture; B. Liver enlargement and congestion; C. There is obvious swelling and bleeding in the intestines; D. The typical symptom of young pigeons with salmonellosis is ‘black belly’, and the whole abdomen is black.

**Figure S2. The results of PCR amplification of 16S rDNA gene of some *Salmonella* isolates.**


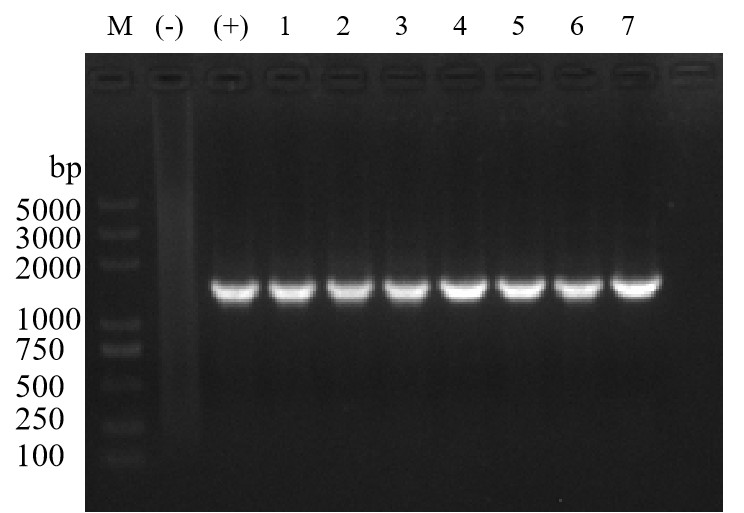


M. DL5000 DNA marker; (-). Negative control; (+). Positive control;1~7. PCR products

**Figure S3. Phenotypic antimicrobial resistance heat map of Salmonella isolates studied.**


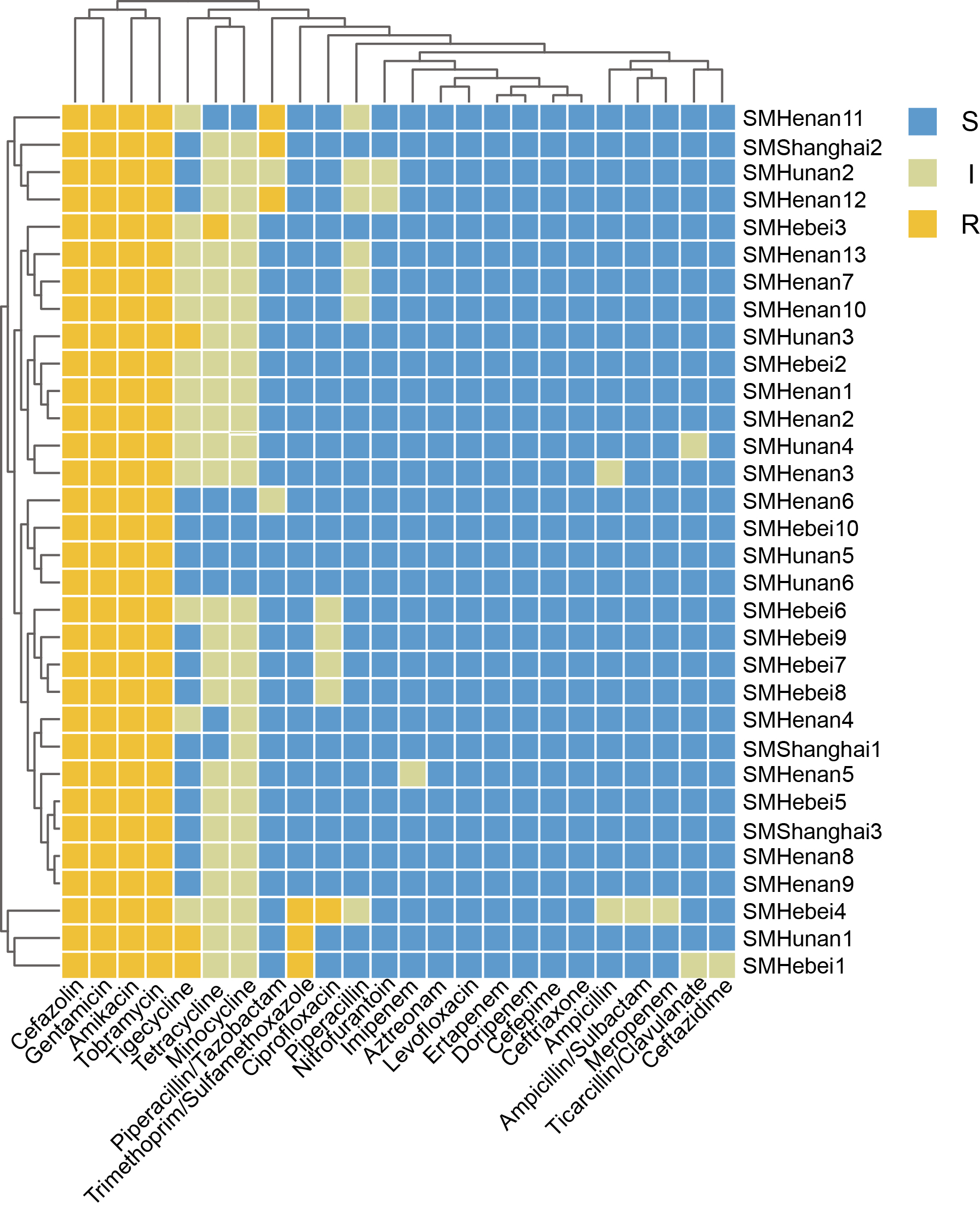


**Table S1. PCR primers used in this study**

| **Primer**  **name** | **Target gene** | **Amplicon**  **size** | **Primer sequence (5’to 3’)** | **PCR condition** |
| --- | --- | --- | --- | --- |
| 27F-  1492R | 16S  rDNA | 1,540bp | F：AGAGTTTGATCCTGGCTCA  R：GGTTACCTTGTTACGACTT | denaturation at 95C for 30 secs annealing at 55℃ for 30 secs and polymerisation  at 72℃ for 60 secs |

27F-1492R Primers was used to identify Salmonella isolates 16S rDNA genes.

**Table S2. Summary of detailed data involved in this study.**

| **Strain** | **Species** | **Provience** | **City** | **Animal** | **ST** | ***aroC*** | ***dnaN*** | ***hemD*** | ***hisD*** | ***purE*** | ***sucA*** | ***thrA*** | **serotype** | **Plasmids** | |
| --- | --- | --- | --- | --- | --- | --- | --- | --- | --- | --- | --- | --- | --- | --- | --- |
|  |  |  |  |  |  |  |  |  |  |  |  |  |  | **IncFIB(S)** | **IncFII(S)** |
| SMHunan1 | *Salmonella* | Hunan | Changde | Meat pigeon | 128 | 10 | 7 | 12 | 9 | 5 | 55 | 2 | Typhimurium O5- (4:i:1,2) | 1 | 1 |
| SMHunan2 | *Salmonella* | Hunan | Changde | Meat pigeon | 128 | 10 | 7 | 12 | 9 | 5 | 55 | 2 | Typhimurium O5- (4:i:1,2) | 1 | 1 |
| SMHunan3 | *Salmonella* | Hunan | Changde | Meat pigeon | 128 | 10 | 7 | 12 | 9 | 5 | 55 | 2 | Typhimurium O5- (4:i:1,2) | 1 | 1 |
| SMHunan4 | *Salmonella* | Hunan | Changde | Meat pigeon | 128 | 10 | 7 | 12 | 9 | 5 | 55 | 2 | Typhimurium O5- (4:i:1,2) | 1 | 1 |
| SMHunan5 | *Salmonella* | Hunan | Changde | Meat pigeon | 128 | 10 | 7 | 12 | 9 | 5 | 55 | 2 | Typhimurium O5- (4:i:1,2) | 1 | 1 |
| SMHunan6 | *Salmonella* | Hunan | Changde | Meat pigeon | 128 | 10 | 7 | 12 | 9 | 5 | 55 | 2 | Typhimurium O5- (4:i:1,2) | 1 | 1 |
| SMHenan1 | *Salmonella* | Henan | Pingdingshan | Meat pigeon | 128 | 10 | 7 | 12 | 9 | 5 | 55 | 2 | Typhimurium O5- (4:i:1,2) | 1 | 1 |
| SMHenan2 | *Salmonella* | Henan | Pingdingshan | Meat pigeon | 128 | 10 | 7 | 12 | 9 | 5 | 55 | 2 | Typhimurium O5- (4:i:1,2) | 1 | 1 |
| SMHenan3 | *Salmonella* | Henan | Pingdingshan | Meat pigeon | 128 | 10 | 7 | 12 | 9 | 5 | 55 | 2 | Typhimurium O5- (4:i:1,2) | 1 | 1 |
| SMHenan4 | *Salmonella* | Henan | Pingdingshan | Meat pigeon | 128 | 10 | 7 | 12 | 9 | 5 | 55 | 2 | Typhimurium O5- (4:i:1,2) | 1 | 1 |
| SMHenan5 | *Salmonella* | Henan | Pingdingshan | Meat pigeon | 128 | 10 | 7 | 12 | 9 | 5 | 55 | 2 | Typhimurium O5- (4:i:1,2) | 1 | 1 |
| SMHebei1 | *Salmonella* | Hebei | Shijiazhuang | Meat pigeon | 128 | 10 | 7 | 12 | 9 | 5 | 55 | 2 | Typhimurium O5- (4:i:1,2) | 1 | 1 |
| SMHebei2 | *Salmonella* | Hebei | Shijiazhuang | Meat pigeon | 128 | 10 | 7 | 12 | 9 | 5 | 55 | 2 | Typhimurium O5- (4:i:1,2) | 1 | 1 |
| SMHebei3 | *Salmonella* | Hebei | Shijiazhuang | Meat pigeon | 128 | 10 | 7 | 12 | 9 | 5 | 55 | 2 | Typhimurium O5- (4:i:1,2) | 1 | 1 |
| SMHebei4 | *Salmonella* | Hebei | Hengshui | Meat pigeon | 128 | 10 | 7 | 12 | 9 | 5 | 55 | 2 | Typhimurium O5- (4:i:1,2) | 1 | 1 |
| SMHebei5 | *Salmonella* | Hebei | Hengshui | Meat pigeon | 128 | 10 | 7 | 12 | 9 | 5 | 55 | 2 | Typhimurium O5- (4:i:1,2) | 1 | 1 |
| SMHebei6 | *Salmonella* | Hebei | Hengshui | Meat pigeon | 128 | 10 | 7 | 12 | 9 | 5 | 55 | 2 | Typhimurium O5- (4:i:1,2) | 1 | 1 |
| SMHebei7 | *Salmonella* | Hebei | Hengshui | Meat pigeon | 128 | 10 | 7 | 12 | 9 | 5 | 55 | 2 | Typhimurium O5- (4:i:1,2) | 1 | 1 |
| SMHebei8 | *Salmonella* | Hebei | Hengshui | Meat pigeon | 128 | 10 | 7 | 12 | 9 | 5 | 55 | 2 | Typhimurium O5- (4:i:1,2) | 1 | 1 |
| SMHebei9 | *Salmonella* | Hebei | Hengshui | Meat pigeon | 128 | 10 | 7 | 12 | 9 | 5 | 55 | 2 | Typhimurium O5- (4:i:1,2) | 1 | 1 |
| SMHebei10 | *Salmonella* | Hebei | Cangzhou | Meat pigeon | 128 | 10 | 7 | 12 | 9 | 5 | 55 | 2 | Typhimurium O5- (4:i:1,2) | 1 | 1 |
| SMShanghai1 | *Salmonella* | Shanghai | Shanghai | Meat pigeon | 128 | 10 | 7 | 12 | 9 | 5 | 55 | 2 | Typhimurium O5- (4:i:1,2) | 1 | 1 |
| SMShanghai2 | *Salmonella* | Shanghai | Shanghai | Meat pigeon | 128 | 10 | 7 | 12 | 9 | 5 | 55 | 2 | Typhimurium O5- (4:i:1,2) | 1 | 1 |
| SMShanghai3 | *Salmonella* | Shanghai | Shanghai | Meat pigeon | 128 | 10 | 7 | 640 | 9 | 5 | 55 | 2 | Typhimurium O5- (4:i:1,2) | 1 | 1 |
| SMHenan6 | *Salmonella* | Henan | Pingdingshan | Meat pigeon | 128 | 10 | 7 | 12 | 9 | 5 | 55 | 2 | Typhimurium O5- (4:i:1,2) | 1 | 1 |
| SMHenan7 | *Salmonella* | Henan | Pingdingshan | Meat pigeon | 128 | 10 | 7 | 12 | 9 | 5 | 55 | 2 | Typhimurium O5- (4:i:1,2) | 1 | 1 |
| SMHenan8 | *Salmonella* | Henan | Pingdingshan | Meat pigeon | 128 | 10 | 7 | 12 | 9 | 5 | 55 | 2 | Typhimurium O5- (4:i:1,2) | 1 | 1 |
| SMHenan9 | *Salmonella* | Henan | Pingdingshan | Meat pigeon | 128 | 10 | 7 | 12 | 9 | 5 | 55 | 2 | Typhimurium O5- (4:i:1,2) | 1 | 1 |
| SMHenan10 | *Salmonella* | Henan | Pingdingshan | Meat pigeon | 128 | 10 | 7 | 12 | 9 | 5 | 55 | 2 | Typhimurium O5- (4:i:1,2) | 1 | 1 |
| SMHenan11 | *Salmonella* | Henan | Pingdingshan | Meat pigeon | 128 | 10 | 7 | 12 | 9 | 5 | 55 | 2 | Typhimurium O5- (4:i:1,2) | 1 | 1 |
| SMHenan12 | *Salmonella* | Henan | Pingdingshan | Meat pigeon | 128 | 10 | 7 | 12 | 9 | 5 | 55 | 2 | Typhimurium O5- (4:i:1,2) | 1 | 1 |
| SMHenan13 | *Salmonella* | Henan | Pingdingshan | Meat pigeon | 128 | 10 | 7 | 12 | 9 | 5 | 55 | 2 | Typhimurium O5- (4:i:1,2) | 1 | 1 |

| **Strain** | **ARGene** | | | | | | | **IS** | | | | | **VDFB** | **Fimbrial adherence determinants** | | | | | | |
| --- | --- | --- | --- | --- | --- | --- | --- | --- | --- | --- | --- | --- | --- | --- | --- | --- | --- | --- | --- | --- |
|  |  |  |  |  |  |  |  |  |  |  |  |  |  | **Agf/Csg** | | | | | | |
|  | ***AAC(6')-Iaa*** | ***golS*** | ***mdsA*** | ***mdsB*** | ***sdiA*** | ***gyrA*(D87N)** | ***gyrA*(S83F)** | **MITEEc1** | **ISEcI10** | **ISSen1** | **ISSen7** | **ISSty2** | **Number-found** | ***csgA*** | ***csgB*** | ***csgC*** | ***csgD*** | ***csgE*** | ***csgF*** | ***csgG*** |
| SMHunan1 | 1 | 1 | 1 | 1 | 1 | 0 | 0 | 1 | 1 | 1 | 1 | 1 | 153 | 1 | 1 | 1 | 1 | 1 | 1 | 1 |
| SMHunan2 | 1 | 1 | 1 | 1 | 1 | 0 | 0 | 1 | 1 | 1 | 1 | 1 | 156 | 1 | 1 | 1 | 1 | 1 | 1 | 1 |
| SMHunan3 | 1 | 1 | 1 | 1 | 1 | 0 | 0 | 1 | 1 | 1 | 1 | 1 | 156 | 1 | 1 | 1 | 1 | 1 | 1 | 1 |
| SMHunan4 | 1 | 1 | 1 | 1 | 1 | 0 | 0 | 1 | 1 | 1 | 1 | 1 | 153 | 1 | 1 | 1 | 1 | 1 | 1 | 1 |
| SMHunan5 | 1 | 1 | 1 | 1 | 1 | 0 | 0 | 1 | 1 | 1 | 1 | 1 | 156 | 1 | 1 | 1 | 1 | 1 | 1 | 1 |
| SMHunan6 | 1 | 1 | 1 | 1 | 1 | 0 | 0 | 1 | 1 | 1 | 1 | 1 | 156 | 1 | 1 | 1 | 1 | 1 | 1 | 1 |
| SMHenan1 | 1 | 1 | 1 | 1 | 1 | 1 | 0 | 1 | 1 | 1 | 1 | 1 | 153 | 1 | 1 | 1 | 1 | 1 | 1 | 1 |
| SMHenan2 | 1 | 1 | 1 | 1 | 1 | 1 | 0 | 1 | 1 | 1 | 1 | 1 | 153 | 1 | 1 | 1 | 1 | 1 | 1 | 1 |
| SMHenan3 | 1 | 1 | 1 | 1 | 1 | 1 | 0 | 1 | 1 | 1 | 1 | 1 | 153 | 1 | 1 | 1 | 1 | 1 | 1 | 1 |
| SMHenan4 | 1 | 1 | 1 | 1 | 1 | 1 | 0 | 1 | 1 | 1 | 1 | 1 | 153 | 1 | 1 | 1 | 1 | 1 | 1 | 1 |
| SMHenan5 | 1 | 1 | 1 | 1 | 1 | 1 | 0 | 1 | 1 | 1 | 1 | 1 | 153 | 1 | 1 | 1 | 1 | 1 | 1 | 1 |
| SMHebei1 | 1 | 1 | 1 | 1 | 1 | 1 | 0 | 1 | 1 | 1 | 1 | 1 | 156 | 1 | 1 | 1 | 1 | 1 | 1 | 1 |
| SMHebei2 | 1 | 1 | 1 | 1 | 1 | 1 | 0 | 1 | 1 | 1 | 1 | 1 | 153 | 1 | 1 | 1 | 1 | 1 | 1 | 1 |
| SMHebei3 | 1 | 1 | 1 | 1 | 1 | 1 | 0 | 1 | 1 | 1 | 1 | 1 | 153 | 1 | 1 | 1 | 1 | 1 | 1 | 1 |
| SMHebei4 | 1 | 1 | 1 | 1 | 1 | 1 | 0 | 1 | 1 | 1 | 1 | 1 | 155 | 1 | 1 | 1 | 1 | 1 | 1 | 1 |
| SMHebei5 | 1 | 1 | 1 | 1 | 1 | 0 | 1 | 1 | 1 | 1 | 1 | 1 | 156 | 1 | 1 | 1 | 1 | 1 | 1 | 1 |
| SMHebei6 | 1 | 1 | 1 | 1 | 1 | 0 | 1 | 1 | 1 | 1 | 1 | 1 | 154 | 1 | 1 | 1 | 1 | 1 | 1 | 1 |
| SMHebei7 | 1 | 1 | 1 | 1 | 1 | 0 | 1 | 1 | 1 | 1 | 1 | 1 | 154 | 1 | 1 | 1 | 1 | 1 | 1 | 1 |
| SMHebei8 | 1 | 1 | 1 | 1 | 1 | 0 | 1 | 1 | 1 | 1 | 1 | 1 | 153 | 1 | 1 | 1 | 1 | 1 | 1 | 1 |
| SMHebei9 | 1 | 1 | 1 | 1 | 1 | 0 | 1 | 1 | 1 | 1 | 1 | 1 | 156 | 1 | 1 | 1 | 1 | 1 | 1 | 1 |
| SMHebei10 | 1 | 1 | 1 | 1 | 1 | 1 | 0 | 1 | 1 | 1 | 1 | 1 | 153 | 1 | 1 | 1 | 1 | 1 | 1 | 1 |
| SMShanghai1 | 1 | 1 | 1 | 1 | 1 | 0 | 1 | 1 | 1 | 1 | 1 | 1 | 152 | 1 | 1 | 1 | 1 | 1 | 1 | 1 |
| SMShanghai2 | 1 | 1 | 1 | 1 | 1 | 0 | 1 | 1 | 1 | 1 | 1 | 1 | 155 | 1 | 1 | 1 | 1 | 1 | 1 | 1 |
| SMShanghai3 | 1 | 1 | 1 | 1 | 1 | 0 | 1 | 1 | 1 | 1 | 1 | 1 | 152 | 1 | 1 | 1 | 1 | 1 | 1 | 1 |
| SMHenan6 | 1 | 1 | 1 | 1 | 1 | 1 | 0 | 1 | 1 | 1 | 1 | 1 | 153 | 1 | 1 | 1 | 1 | 1 | 1 | 1 |
| SMHenan7 | 1 | 1 | 1 | 1 | 1 | 1 | 0 | 1 | 1 | 1 | 1 | 1 | 155 | 1 | 1 | 1 | 1 | 1 | 1 | 1 |
| SMHenan8 | 1 | 1 | 1 | 1 | 1 | 1 | 0 | 1 | 1 | 1 | 1 | 1 | 153 | 1 | 1 | 1 | 1 | 1 | 1 | 1 |
| SMHenan9 | 1 | 1 | 1 | 1 | 1 | 1 | 0 | 1 | 1 | 1 | 1 | 1 | 153 | 1 | 1 | 1 | 1 | 1 | 1 | 1 |
| SMHenan10 | 1 | 1 | 1 | 1 | 1 | 1 | 0 | 1 | 1 | 1 | 1 | 1 | 153 | 1 | 1 | 1 | 1 | 1 | 1 | 1 |
| SMHenan11 | 1 | 1 | 1 | 1 | 1 | 1 | 0 | 1 | 1 | 1 | 1 | 1 | 152 | 1 | 1 | 1 | 1 | 1 | 1 | 1 |
| SMHenan12 | 1 | 1 | 1 | 1 | 1 | 1 | 0 | 1 | 1 | 1 | 1 | 1 | 153 | 1 | 1 | 1 | 1 | 1 | 1 | 1 |
| SMHenan13 | 1 | 1 | 1 | 1 | 1 | 1 | 0 | 1 | 1 | 1 | 1 | 1 | 153 | 1 | 1 | 1 | 1 | 1 | 1 | 1 |

| **Strain** | **Fimbrial adherence determinants** | | | | | | | | | | | | | | | | | | | | | | | | |
| --- | --- | --- | --- | --- | --- | --- | --- | --- | --- | --- | --- | --- | --- | --- | --- | --- | --- | --- | --- | --- | --- | --- | --- | --- | --- |
|  | **Bcf** | | | | | | | **Fim** | | | | | | | | | **Lpf** | | | | | **Pef** | | | |
|  | ***bcfA*** | ***bcfB*** | ***bcfC*** | ***bcfD*** | ***bcfE*** | ***bcfF*** | ***bcfG*** | ***fimA*** | ***fimC*** | ***fimD*** | ***fimF*** | ***fimH*** | ***fimI*** | ***fimW*** | ***fimY*** | ***fimZ*** | ***lpfA*** | ***lpfB*** | ***lpfC*** | ***lpfD*** | ***lpfE*** | ***pefA*** | ***pefB*** | ***pefC*** | ***pefD*** |
| SMHunan1 | 1 | 1 | 1 | 1 | 1 | 1 | 1 | 1 | 1 | 1 | 1 | 1 | 1 | 1 | 1 | 1 | 1 | 1 | 1 | 1 | 1 | 1 | 1 | 1 | 1 |
| SMHunan2 | 1 | 1 | 1 | 1 | 1 | 1 | 1 | 1 | 1 | 1 | 1 | 1 | 1 | 1 | 1 | 1 | 1 | 1 | 1 | 1 | 1 | 1 | 1 | 1 | 1 |
| SMHunan3 | 1 | 1 | 1 | 1 | 1 | 1 | 1 | 1 | 1 | 1 | 1 | 1 | 1 | 1 | 1 | 1 | 1 | 1 | 1 | 1 | 1 | 1 | 1 | 1 | 1 |
| SMHunan4 | 1 | 1 | 1 | 1 | 1 | 1 | 1 | 1 | 1 | 1 | 1 | 1 | 1 | 1 | 1 | 1 | 1 | 1 | 1 | 1 | 1 | 1 | 1 | 1 | 1 |
| SMHunan5 | 1 | 1 | 1 | 1 | 1 | 1 | 1 | 1 | 1 | 1 | 1 | 1 | 1 | 1 | 1 | 1 | 1 | 1 | 1 | 1 | 1 | 1 | 1 | 1 | 1 |
| SMHunan6 | 1 | 1 | 1 | 1 | 1 | 1 | 1 | 1 | 1 | 1 | 1 | 1 | 1 | 1 | 1 | 1 | 1 | 1 | 1 | 1 | 1 | 1 | 1 | 1 | 1 |
| SMHenan1 | 1 | 1 | 1 | 1 | 1 | 1 | 1 | 1 | 1 | 1 | 1 | 1 | 1 | 1 | 1 | 1 | 1 | 1 | 1 | 1 | 1 | 1 | 1 | 1 | 1 |
| SMHenan2 | 1 | 1 | 1 | 1 | 1 | 1 | 1 | 1 | 1 | 1 | 1 | 1 | 1 | 1 | 1 | 1 | 1 | 1 | 1 | 1 | 1 | 1 | 1 | 1 | 1 |
| SMHenan3 | 1 | 1 | 1 | 1 | 1 | 1 | 1 | 1 | 1 | 1 | 1 | 1 | 1 | 1 | 1 | 1 | 1 | 1 | 1 | 1 | 1 | 1 | 1 | 1 | 1 |
| SMHenan4 | 1 | 1 | 1 | 1 | 1 | 1 | 1 | 1 | 1 | 1 | 1 | 1 | 1 | 1 | 1 | 1 | 1 | 1 | 1 | 1 | 1 | 1 | 1 | 1 | 1 |
| SMHenan5 | 1 | 1 | 1 | 1 | 1 | 1 | 1 | 1 | 1 | 1 | 1 | 1 | 1 | 1 | 1 | 1 | 1 | 1 | 1 | 1 | 1 | 1 | 1 | 1 | 1 |
| SMHebei1 | 1 | 1 | 1 | 1 | 1 | 1 | 1 | 1 | 1 | 1 | 1 | 1 | 1 | 1 | 1 | 1 | 1 | 1 | 1 | 1 | 1 | 1 | 1 | 1 | 1 |
| SMHebei2 | 1 | 1 | 1 | 1 | 1 | 1 | 1 | 1 | 1 | 1 | 1 | 1 | 1 | 1 | 1 | 1 | 1 | 1 | 1 | 1 | 1 | 1 | 1 | 1 | 1 |
| SMHebei3 | 1 | 1 | 1 | 1 | 1 | 1 | 1 | 1 | 1 | 1 | 1 | 1 | 1 | 1 | 1 | 1 | 1 | 1 | 1 | 1 | 1 | 1 | 1 | 1 | 1 |
| SMHebei4 | 1 | 1 | 1 | 1 | 1 | 1 | 1 | 1 | 1 | 1 | 1 | 1 | 1 | 1 | 1 | 1 | 1 | 1 | 1 | 1 | 1 | 1 | 1 | 1 | 1 |
| SMHebei5 | 1 | 1 | 1 | 1 | 1 | 1 | 1 | 1 | 1 | 1 | 1 | 1 | 1 | 1 | 1 | 1 | 1 | 1 | 1 | 1 | 1 | 1 | 1 | 1 | 1 |
| SMHebei6 | 1 | 1 | 1 | 1 | 1 | 1 | 1 | 1 | 1 | 1 | 1 | 1 | 1 | 1 | 1 | 1 | 1 | 1 | 1 | 1 | 1 | 1 | 1 | 1 | 1 |
| SMHebei7 | 1 | 1 | 1 | 1 | 1 | 1 | 1 | 1 | 1 | 1 | 1 | 1 | 1 | 1 | 1 | 1 | 1 | 1 | 1 | 1 | 1 | 1 | 1 | 1 | 1 |
| SMHebei8 | 1 | 1 | 1 | 1 | 1 | 1 | 1 | 1 | 1 | 1 | 1 | 1 | 1 | 1 | 1 | 1 | 1 | 1 | 1 | 1 | 1 | 1 | 1 | 1 | 1 |
| SMHebei9 | 1 | 1 | 1 | 1 | 1 | 1 | 1 | 1 | 1 | 1 | 1 | 1 | 1 | 1 | 1 | 1 | 1 | 1 | 1 | 1 | 1 | 1 | 1 | 1 | 1 |
| SMHebei10 | 1 | 1 | 1 | 1 | 1 | 1 | 1 | 1 | 1 | 1 | 1 | 1 | 1 | 1 | 1 | 1 | 1 | 1 | 1 | 1 | 1 | 1 | 1 | 1 | 1 |
| SMShanghai1 | 1 | 1 | 1 | 1 | 1 | 1 | 1 | 1 | 1 | 1 | 1 | 1 | 1 | 1 | 1 | 1 | 1 | 1 | 1 | 1 | 1 | 1 | 1 | 1 | 1 |
| SMShanghai2 | 1 | 1 | 1 | 1 | 1 | 1 | 1 | 1 | 1 | 1 | 1 | 1 | 1 | 1 | 1 | 1 | 1 | 1 | 1 | 1 | 1 | 1 | 1 | 1 | 1 |
| SMShanghai3 | 1 | 1 | 1 | 1 | 1 | 1 | 1 | 1 | 1 | 1 | 1 | 1 | 1 | 1 | 1 | 1 | 1 | 1 | 1 | 1 | 1 | 1 | 1 | 1 | 1 |
| SMHenan6 | 1 | 1 | 1 | 1 | 1 | 1 | 1 | 1 | 1 | 1 | 1 | 1 | 1 | 1 | 1 | 1 | 1 | 1 | 1 | 1 | 1 | 1 | 1 | 1 | 1 |
| SMHenan7 | 1 | 1 | 1 | 1 | 1 | 1 | 1 | 1 | 1 | 1 | 1 | 1 | 1 | 1 | 1 | 1 | 1 | 1 | 1 | 1 | 1 | 1 | 1 | 1 | 1 |
| SMHenan8 | 1 | 1 | 1 | 1 | 1 | 1 | 1 | 1 | 1 | 1 | 1 | 1 | 1 | 1 | 1 | 1 | 1 | 1 | 1 | 1 | 1 | 1 | 1 | 1 | 1 |
| SMHenan9 | 1 | 1 | 1 | 1 | 1 | 1 | 1 | 1 | 1 | 1 | 1 | 1 | 1 | 1 | 1 | 1 | 1 | 1 | 1 | 1 | 1 | 1 | 1 | 1 | 1 |
| SMHenan10 | 1 | 1 | 1 | 1 | 1 | 1 | 1 | 1 | 1 | 1 | 1 | 1 | 1 | 1 | 1 | 1 | 1 | 1 | 1 | 1 | 1 | 1 | 1 | 1 | 1 |
| SMHenan11 | 1 | 1 | 1 | 1 | 1 | 1 | 1 | 1 | 1 | 1 | 1 | 1 | 1 | 1 | 1 | 1 | 1 | 1 | 1 | 1 | 1 | 1 | 1 | 1 | 1 |
| SMHenan12 | 1 | 1 | 1 | 1 | 1 | 1 | 1 | 1 | 1 | 1 | 1 | 1 | 1 | 1 | 1 | 1 | 1 | 1 | 1 | 1 | 1 | 1 | 1 | 1 | 1 |
| SMHenan13 | 1 | 1 | 1 | 1 | 1 | 1 | 1 | 1 | 1 | 1 | 1 | 1 | 1 | 1 | 1 | 1 | 1 | 1 | 1 | 1 | 1 | 1 | 1 | 1 | 1 |

| **Strain** | **Fimbrial adherence determinants** | | | | | | | | | | | | | | | | | | | | | | | | | |
| --- | --- | --- | --- | --- | --- | --- | --- | --- | --- | --- | --- | --- | --- | --- | --- | --- | --- | --- | --- | --- | --- | --- | --- | --- | --- | --- |
|  | **Saf** | | | **Stb** | | | | | **Stc** | | | | **Std** | | | **Stf** | | | | | | **Sth** | | | | |
|  | ***safB*** | ***safC*** | ***safD*** | ***stbA*** | ***stbB*** | ***stbC*** | ***stbD*** | ***stbE*** | ***stcA*** | ***stcB*** | ***stcC*** | ***stcD*** | ***stdA*** | ***stdB*** | ***stdC*** | ***stfA*** | ***stfC*** | ***stfD*** | ***stfE*** | ***stfF*** | ***stfG*** | ***sthA*** | ***sthB*** | ***sthC*** | ***sthD*** | ***sthE*** |
| SMHunan1 | 1 | 1 | 0 | 1 | 1 | 1 | 1 | 1 | 1 | 1 | 1 | 1 | 1 | 1 | 1 | 1 | 1 | 1 | 1 | 1 | 1 | 1 | 1 | 1 | 1 | 1 |
| SMHunan2 | 1 | 1 | 1 | 1 | 1 | 1 | 1 | 1 | 1 | 1 | 1 | 1 | 1 | 1 | 1 | 1 | 1 | 1 | 1 | 1 | 1 | 1 | 1 | 1 | 1 | 1 |
| SMHunan3 | 1 | 1 | 1 | 1 | 1 | 1 | 1 | 1 | 1 | 1 | 1 | 1 | 1 | 1 | 1 | 1 | 1 | 1 | 1 | 1 | 1 | 1 | 1 | 1 | 1 | 1 |
| SMHunan4 | 1 | 1 | 0 | 1 | 1 | 1 | 1 | 1 | 1 | 1 | 1 | 1 | 1 | 1 | 1 | 1 | 1 | 1 | 1 | 1 | 1 | 1 | 1 | 1 | 1 | 1 |
| SMHunan5 | 1 | 1 | 1 | 1 | 1 | 1 | 1 | 1 | 1 | 1 | 1 | 1 | 1 | 1 | 1 | 1 | 1 | 1 | 1 | 1 | 1 | 1 | 1 | 1 | 1 | 1 |
| SMHunan6 | 1 | 1 | 1 | 1 | 1 | 1 | 1 | 1 | 1 | 1 | 1 | 1 | 1 | 1 | 1 | 1 | 1 | 1 | 1 | 1 | 1 | 1 | 1 | 1 | 1 | 1 |
| SMHenan1 | 1 | 1 | 1 | 1 | 1 | 1 | 1 | 1 | 1 | 1 | 1 | 1 | 1 | 1 | 0 | 1 | 1 | 1 | 1 | 1 | 1 | 1 | 1 | 1 | 1 | 1 |
| SMHenan2 | 1 | 1 | 1 | 1 | 1 | 1 | 1 | 1 | 1 | 1 | 1 | 1 | 1 | 1 | 0 | 1 | 1 | 1 | 1 | 1 | 1 | 1 | 1 | 1 | 1 | 1 |
| SMHenan3 | 1 | 1 | 1 | 1 | 1 | 1 | 1 | 1 | 1 | 1 | 1 | 1 | 1 | 1 | 0 | 1 | 1 | 1 | 1 | 1 | 1 | 1 | 1 | 1 | 1 | 1 |
| SMHenan4 | 1 | 1 | 1 | 1 | 1 | 1 | 1 | 1 | 1 | 1 | 1 | 1 | 1 | 1 | 0 | 1 | 1 | 1 | 1 | 1 | 1 | 1 | 1 | 1 | 1 | 1 |
| SMHenan5 | 1 | 1 | 1 | 1 | 1 | 1 | 1 | 1 | 1 | 1 | 1 | 1 | 1 | 1 | 0 | 1 | 1 | 1 | 1 | 1 | 1 | 1 | 1 | 1 | 1 | 1 |
| SMHebei1 | 1 | 1 | 1 | 1 | 1 | 1 | 1 | 1 | 1 | 1 | 1 | 1 | 1 | 1 | 1 | 1 | 1 | 1 | 1 | 1 | 1 | 1 | 1 | 1 | 1 | 1 |
| SMHebei2 | 1 | 1 | 0 | 1 | 1 | 1 | 1 | 1 | 1 | 1 | 1 | 1 | 1 | 1 | 1 | 1 | 1 | 1 | 1 | 1 | 1 | 1 | 1 | 1 | 1 | 1 |
| SMHebei3 | 1 | 1 | 0 | 1 | 1 | 1 | 1 | 1 | 1 | 1 | 1 | 1 | 1 | 1 | 1 | 1 | 1 | 1 | 1 | 1 | 1 | 1 | 1 | 1 | 1 | 1 |
| SMHebei4 | 1 | 1 | 1 | 1 | 1 | 1 | 1 | 1 | 1 | 1 | 1 | 1 | 1 | 1 | 0 | 1 | 1 | 1 | 1 | 1 | 1 | 1 | 1 | 1 | 1 | 1 |
| SMHebei5 | 1 | 1 | 1 | 1 | 1 | 1 | 1 | 1 | 1 | 1 | 1 | 1 | 1 | 1 | 1 | 1 | 1 | 1 | 1 | 1 | 1 | 1 | 1 | 1 | 1 | 1 |
| SMHebei6 | 1 | 1 | 1 | 1 | 1 | 1 | 1 | 1 | 1 | 1 | 1 | 1 | 1 | 1 | 1 | 1 | 1 | 1 | 1 | 1 | 1 | 1 | 1 | 1 | 1 | 1 |
| SMHebei7 | 1 | 1 | 0 | 1 | 1 | 1 | 1 | 1 | 1 | 1 | 1 | 1 | 1 | 1 | 1 | 1 | 1 | 1 | 1 | 1 | 1 | 1 | 1 | 1 | 1 | 1 |
| SMHebei8 | 1 | 1 | 1 | 1 | 1 | 1 | 1 | 1 | 1 | 1 | 1 | 1 | 1 | 1 | 1 | 1 | 1 | 1 | 1 | 1 | 1 | 1 | 1 | 1 | 1 | 1 |
| SMHebei9 | 1 | 1 | 1 | 1 | 1 | 1 | 1 | 1 | 1 | 1 | 1 | 1 | 1 | 1 | 1 | 1 | 1 | 1 | 1 | 1 | 1 | 1 | 1 | 1 | 1 | 1 |
| SMHebei10 | 1 | 1 | 1 | 1 | 1 | 1 | 1 | 1 | 1 | 1 | 1 | 1 | 1 | 1 | 0 | 1 | 1 | 1 | 1 | 1 | 1 | 1 | 1 | 1 | 1 | 1 |
| SMShanghai1 | 1 | 1 | 1 | 1 | 1 | 1 | 1 | 1 | 1 | 1 | 1 | 1 | 1 | 1 | 0 | 1 | 1 | 1 | 1 | 1 | 1 | 1 | 1 | 1 | 1 | 1 |
| SMShanghai2 | 1 | 1 | 1 | 1 | 1 | 1 | 1 | 1 | 1 | 1 | 1 | 1 | 1 | 1 | 0 | 1 | 1 | 1 | 1 | 1 | 1 | 1 | 1 | 1 | 1 | 1 |
| SMShanghai3 | 1 | 1 | 1 | 1 | 1 | 1 | 1 | 1 | 1 | 1 | 1 | 1 | 1 | 1 | 0 | 1 | 1 | 1 | 1 | 1 | 1 | 1 | 1 | 1 | 1 | 1 |
| SMHenan6 | 1 | 1 | 1 | 1 | 1 | 1 | 1 | 1 | 1 | 1 | 1 | 1 | 1 | 1 | 0 | 1 | 1 | 1 | 1 | 1 | 1 | 1 | 1 | 1 | 1 | 1 |
| SMHenan7 | 1 | 1 | 1 | 1 | 1 | 1 | 1 | 1 | 1 | 1 | 1 | 1 | 1 | 1 | 0 | 1 | 1 | 1 | 1 | 1 | 1 | 1 | 1 | 1 | 1 | 1 |
| SMHenan8 | 1 | 1 | 1 | 1 | 1 | 1 | 1 | 1 | 1 | 1 | 1 | 1 | 1 | 1 | 0 | 1 | 1 | 1 | 1 | 1 | 1 | 1 | 1 | 1 | 1 | 1 |
| SMHenan9 | 1 | 1 | 1 | 1 | 1 | 1 | 1 | 1 | 1 | 1 | 1 | 1 | 1 | 1 | 0 | 1 | 1 | 1 | 1 | 1 | 1 | 1 | 1 | 1 | 1 | 1 |
| SMHenan10 | 1 | 1 | 1 | 1 | 1 | 1 | 1 | 1 | 1 | 1 | 1 | 1 | 1 | 1 | 0 | 1 | 1 | 1 | 1 | 1 | 1 | 1 | 1 | 1 | 1 | 1 |
| SMHenan11 | 1 | 1 | 0 | 1 | 1 | 1 | 1 | 1 | 1 | 1 | 1 | 1 | 1 | 1 | 0 | 1 | 1 | 1 | 1 | 1 | 1 | 1 | 1 | 1 | 1 | 1 |
| SMHenan12 | 1 | 1 | 1 | 1 | 1 | 1 | 1 | 1 | 1 | 1 | 1 | 1 | 1 | 1 | 0 | 1 | 1 | 1 | 1 | 1 | 1 | 1 | 1 | 1 | 1 | 1 |
| SMHenan13 | 1 | 1 | 1 | 1 | 1 | 1 | 1 | 1 | 1 | 1 | 1 | 1 | 1 | 1 | 0 | 1 | 1 | 1 | 1 | 1 | 1 | 1 | 1 | 1 | 1 | 1 |

| **Strain** | **Fimbrial adherence determinants** | | | | | | **Macrophage inducible genes** | | **Magnesium uptake** | | **Nonfimbrial adherence determinants** | | | | **Regulation** | | **Secretion system** | | | | |
| --- | --- | --- | --- | --- | --- | --- | --- | --- | --- | --- | --- | --- | --- | --- | --- | --- | --- | --- | --- | --- | --- |
|  | **Sti** | | | | **Stj** | | **Mig-14** | **Mig-5** | **Mg2+ transport** | | **MisL** | **RatB** | **ShdA** | **SinH** | **PhoPQ** | | **TTSS (SPI-1 encode)** | | | | |
|  | ***stiA*** | ***stiB*** | ***stiC*** | ***stiH*** | ***stjB*** | ***stjC*** | ***mig-14*** | ***mig-5*** | ***mgtB*** | ***mgtC*** | ***misL*** | ***ratB*** | ***shdA*** | ***sinH*** | ***phoP*** | ***phoQ*** | ***hilA*** | ***hilC*** | ***hilD*** | ***iacP*** | ***iagB*** |
| SMHunan1 | 1 | 1 | 1 | 1 | 1 | 1 | 1 | 1 | 1 | 1 | 1 | 1 | 1 | 1 | 1 | 1 | 1 | 1 | 1 | 1 | 1 |
| SMHunan2 | 1 | 1 | 1 | 1 | 1 | 1 | 1 | 1 | 1 | 1 | 1 | 1 | 1 | 1 | 1 | 1 | 1 | 1 | 1 | 1 | 1 |
| SMHunan3 | 1 | 1 | 1 | 1 | 1 | 1 | 1 | 1 | 1 | 1 | 1 | 1 | 1 | 1 | 1 | 1 | 1 | 1 | 1 | 1 | 1 |
| SMHunan4 | 1 | 1 | 1 | 1 | 1 | 1 | 1 | 1 | 1 | 1 | 1 | 1 | 1 | 1 | 1 | 1 | 1 | 1 | 1 | 1 | 1 |
| SMHunan5 | 1 | 1 | 1 | 1 | 1 | 1 | 1 | 1 | 1 | 1 | 1 | 1 | 1 | 1 | 1 | 1 | 1 | 1 | 1 | 1 | 1 |
| SMHunan6 | 1 | 1 | 1 | 1 | 1 | 1 | 1 | 1 | 1 | 1 | 1 | 1 | 1 | 1 | 1 | 1 | 1 | 1 | 1 | 1 | 1 |
| SMHenan1 | 1 | 1 | 1 | 1 | 1 | 1 | 1 | 1 | 1 | 1 | 1 | 1 | 1 | 1 | 1 | 1 | 1 | 1 | 1 | 1 | 1 |
| SMHenan2 | 1 | 1 | 1 | 1 | 1 | 1 | 1 | 1 | 1 | 1 | 1 | 1 | 1 | 1 | 1 | 1 | 1 | 1 | 1 | 1 | 1 |
| SMHenan3 | 1 | 1 | 1 | 1 | 1 | 1 | 1 | 1 | 1 | 1 | 1 | 1 | 1 | 1 | 1 | 1 | 1 | 1 | 1 | 1 | 1 |
| SMHenan4 | 1 | 1 | 1 | 1 | 1 | 1 | 1 | 1 | 1 | 1 | 1 | 1 | 1 | 1 | 1 | 1 | 1 | 1 | 1 | 1 | 1 |
| SMHenan5 | 1 | 1 | 1 | 1 | 1 | 1 | 1 | 1 | 1 | 1 | 1 | 1 | 1 | 1 | 1 | 1 | 1 | 1 | 1 | 1 | 1 |
| SMHebei1 | 1 | 1 | 1 | 1 | 1 | 1 | 1 | 1 | 1 | 1 | 1 | 1 | 1 | 1 | 1 | 1 | 1 | 1 | 1 | 1 | 1 |
| SMHebei2 | 1 | 1 | 1 | 1 | 1 | 1 | 1 | 1 | 1 | 1 | 1 | 1 | 1 | 1 | 1 | 1 | 1 | 1 | 1 | 1 | 1 |
| SMHebei3 | 1 | 1 | 1 | 1 | 1 | 1 | 1 | 1 | 1 | 1 | 1 | 1 | 1 | 1 | 1 | 1 | 1 | 1 | 1 | 1 | 1 |
| SMHebei4 | 1 | 1 | 1 | 1 | 1 | 1 | 1 | 1 | 1 | 1 | 1 | 1 | 1 | 1 | 1 | 1 | 1 | 1 | 1 | 1 | 1 |
| SMHebei5 | 1 | 1 | 1 | 1 | 1 | 1 | 1 | 1 | 1 | 1 | 1 | 1 | 1 | 1 | 1 | 1 | 1 | 1 | 1 | 1 | 1 |
| SMHebei6 | 1 | 1 | 1 | 1 | 1 | 1 | 1 | 1 | 1 | 1 | 1 | 1 | 1 | 1 | 1 | 1 | 1 | 1 | 1 | 1 | 1 |
| SMHebei7 | 1 | 1 | 1 | 1 | 1 | 1 | 1 | 1 | 1 | 1 | 1 | 1 | 1 | 1 | 1 | 1 | 1 | 1 | 1 | 1 | 1 |
| SMHebei8 | 1 | 1 | 1 | 1 | 1 | 1 | 1 | 1 | 1 | 1 | 1 | 1 | 1 | 1 | 1 | 1 | 1 | 1 | 1 | 1 | 1 |
| SMHebei9 | 1 | 1 | 1 | 1 | 1 | 1 | 1 | 1 | 1 | 1 | 1 | 1 | 1 | 1 | 1 | 1 | 1 | 1 | 1 | 1 | 1 |
| SMHebei10 | 1 | 1 | 1 | 1 | 1 | 1 | 1 | 1 | 1 | 1 | 1 | 1 | 1 | 1 | 1 | 1 | 1 | 1 | 1 | 1 | 1 |
| SMShanghai1 | 1 | 1 | 1 | 1 | 1 | 1 | 1 | 1 | 1 | 1 | 1 | 1 | 1 | 1 | 1 | 1 | 1 | 1 | 1 | 1 | 1 |
| SMShanghai2 | 1 | 1 | 1 | 1 | 1 | 1 | 1 | 1 | 1 | 1 | 1 | 1 | 1 | 1 | 1 | 1 | 1 | 1 | 1 | 1 | 1 |
| SMShanghai3 | 1 | 1 | 1 | 1 | 1 | 1 | 1 | 1 | 1 | 1 | 1 | 1 | 1 | 1 | 1 | 1 | 1 | 1 | 1 | 1 | 1 |
| SMHenan6 | 1 | 1 | 1 | 1 | 1 | 1 | 1 | 1 | 1 | 1 | 1 | 1 | 1 | 1 | 1 | 1 | 1 | 1 | 1 | 1 | 1 |
| SMHenan7 | 1 | 1 | 1 | 1 | 1 | 1 | 1 | 1 | 1 | 1 | 1 | 1 | 1 | 1 | 1 | 1 | 1 | 1 | 1 | 1 | 1 |
| SMHenan8 | 1 | 1 | 1 | 1 | 1 | 1 | 1 | 1 | 1 | 1 | 1 | 1 | 1 | 1 | 1 | 1 | 1 | 1 | 1 | 1 | 1 |
| SMHenan9 | 1 | 1 | 1 | 1 | 1 | 1 | 1 | 1 | 1 | 1 | 1 | 1 | 1 | 1 | 1 | 1 | 1 | 1 | 1 | 1 | 1 |
| SMHenan10 | 1 | 1 | 1 | 1 | 1 | 1 | 1 | 1 | 1 | 1 | 1 | 1 | 1 | 1 | 1 | 1 | 1 | 1 | 1 | 1 | 1 |
| SMHenan11 | 1 | 1 | 1 | 1 | 1 | 1 | 1 | 1 | 1 | 1 | 1 | 1 | 1 | 1 | 1 | 1 | 1 | 1 | 1 | 1 | 1 |
| SMHenan12 | 1 | 1 | 1 | 1 | 1 | 1 | 1 | 1 | 1 | 1 | 1 | 1 | 1 | 1 | 1 | 1 | 1 | 1 | 1 | 1 | 1 |
| SMHenan13 | 1 | 1 | 1 | 1 | 1 | 1 | 1 | 1 | 1 | 1 | 1 | 1 | 1 | 1 | 1 | 1 | 1 | 1 | 1 | 1 | 1 |

| **Strain** | **Secretion system** | | | | | | | | | | | | | | | | | | | | |
| --- | --- | --- | --- | --- | --- | --- | --- | --- | --- | --- | --- | --- | --- | --- | --- | --- | --- | --- | --- | --- | --- |
|  | **TTSS (SPI-1 encode)** | | | | | | | | | | | | | | | | | | | | |
|  | ***invA*** | ***invB*** | ***invC*** | ***invE*** | ***invF*** | ***invG*** | ***invH*** | ***invI*** | ***invJ*** | ***orgA*** | ***orgB*** | ***orgC*** | ***sicA*** | ***sicP*** | ***sipD*** | ***spaO*** | ***spaP*** | ***spaQ*** | ***spaR*** | ***spaS*** | ***sprB*** |
| SMHunan1 | 1 | 1 | 1 | 1 | 1 | 1 | 1 | 1 | 1 | 1 | 1 | 1 | 1 | 1 | 1 | 1 | 1 | 1 | 1 | 1 | 1 |
| SMHunan2 | 1 | 1 | 1 | 1 | 1 | 1 | 1 | 1 | 1 | 1 | 1 | 1 | 1 | 1 | 1 | 1 | 1 | 1 | 1 | 1 | 1 |
| SMHunan3 | 1 | 1 | 1 | 1 | 1 | 1 | 1 | 1 | 1 | 1 | 1 | 1 | 1 | 1 | 1 | 1 | 1 | 1 | 1 | 1 | 1 |
| SMHunan4 | 1 | 1 | 1 | 1 | 1 | 1 | 1 | 1 | 1 | 1 | 1 | 1 | 1 | 1 | 1 | 1 | 1 | 1 | 1 | 1 | 1 |
| SMHunan5 | 1 | 1 | 1 | 1 | 1 | 1 | 1 | 1 | 1 | 1 | 1 | 1 | 1 | 1 | 1 | 1 | 1 | 1 | 1 | 1 | 1 |
| SMHunan6 | 1 | 1 | 1 | 1 | 1 | 1 | 1 | 1 | 1 | 1 | 1 | 1 | 1 | 1 | 1 | 1 | 1 | 1 | 1 | 1 | 1 |
| SMHenan1 | 1 | 1 | 1 | 1 | 1 | 1 | 1 | 1 | 1 | 1 | 1 | 1 | 1 | 1 | 1 | 1 | 1 | 1 | 1 | 1 | 1 |
| SMHenan2 | 1 | 1 | 1 | 1 | 1 | 1 | 1 | 1 | 1 | 1 | 1 | 1 | 1 | 1 | 1 | 1 | 1 | 1 | 1 | 1 | 1 |
| SMHenan3 | 1 | 1 | 1 | 1 | 1 | 1 | 1 | 1 | 1 | 1 | 1 | 1 | 1 | 1 | 1 | 1 | 1 | 1 | 1 | 1 | 1 |
| SMHenan4 | 1 | 1 | 1 | 1 | 1 | 1 | 1 | 1 | 1 | 1 | 1 | 1 | 1 | 1 | 1 | 1 | 1 | 1 | 1 | 1 | 1 |
| SMHenan5 | 1 | 1 | 1 | 1 | 1 | 1 | 1 | 1 | 1 | 1 | 1 | 1 | 1 | 1 | 1 | 1 | 1 | 1 | 1 | 1 | 1 |
| SMHebei1 | 1 | 1 | 1 | 1 | 1 | 1 | 1 | 1 | 1 | 1 | 1 | 1 | 1 | 1 | 1 | 1 | 1 | 1 | 1 | 1 | 1 |
| SMHebei2 | 1 | 1 | 1 | 1 | 1 | 1 | 1 | 1 | 1 | 1 | 1 | 1 | 1 | 1 | 1 | 1 | 1 | 1 | 1 | 1 | 1 |
| SMHebei3 | 1 | 1 | 1 | 1 | 1 | 1 | 1 | 1 | 1 | 1 | 1 | 1 | 1 | 1 | 1 | 1 | 1 | 1 | 1 | 1 | 1 |
| SMHebei4 | 1 | 1 | 1 | 1 | 1 | 1 | 1 | 1 | 1 | 1 | 1 | 1 | 1 | 1 | 1 | 1 | 1 | 1 | 1 | 1 | 1 |
| SMHebei5 | 1 | 1 | 1 | 1 | 1 | 1 | 1 | 1 | 1 | 1 | 1 | 1 | 1 | 1 | 1 | 1 | 1 | 1 | 1 | 1 | 1 |
| SMHebei6 | 1 | 1 | 1 | 1 | 1 | 1 | 1 | 1 | 1 | 1 | 1 | 1 | 1 | 1 | 1 | 1 | 1 | 1 | 1 | 1 | 1 |
| SMHebei7 | 1 | 1 | 1 | 1 | 1 | 1 | 1 | 1 | 1 | 1 | 1 | 1 | 1 | 1 | 1 | 1 | 1 | 1 | 1 | 1 | 1 |
| SMHebei8 | 1 | 1 | 1 | 1 | 1 | 1 | 1 | 1 | 1 | 1 | 1 | 1 | 1 | 1 | 1 | 1 | 1 | 1 | 1 | 1 | 1 |
| SMHebei9 | 1 | 1 | 1 | 1 | 1 | 1 | 1 | 1 | 1 | 1 | 1 | 1 | 1 | 1 | 1 | 1 | 1 | 1 | 1 | 1 | 1 |
| SMHebei10 | 1 | 1 | 1 | 1 | 1 | 1 | 1 | 1 | 1 | 1 | 1 | 1 | 1 | 1 | 1 | 1 | 1 | 1 | 1 | 1 | 1 |
| SMShanghai1 | 1 | 1 | 1 | 1 | 1 | 1 | 1 | 1 | 1 | 1 | 1 | 1 | 1 | 1 | 1 | 1 | 1 | 1 | 1 | 1 | 1 |
| SMShanghai2 | 1 | 1 | 1 | 1 | 1 | 1 | 1 | 1 | 1 | 1 | 1 | 1 | 1 | 1 | 1 | 1 | 1 | 1 | 1 | 1 | 1 |
| SMShanghai3 | 1 | 1 | 1 | 1 | 1 | 1 | 1 | 1 | 1 | 1 | 1 | 1 | 1 | 1 | 1 | 1 | 1 | 1 | 1 | 1 | 1 |
| SMHenan6 | 1 | 1 | 1 | 1 | 1 | 1 | 1 | 1 | 1 | 1 | 1 | 1 | 1 | 1 | 1 | 1 | 1 | 1 | 1 | 1 | 1 |
| SMHenan7 | 1 | 1 | 1 | 1 | 1 | 1 | 1 | 1 | 1 | 1 | 1 | 1 | 1 | 1 | 1 | 1 | 1 | 1 | 1 | 1 | 1 |
| SMHenan8 | 1 | 1 | 1 | 1 | 1 | 1 | 1 | 1 | 1 | 1 | 1 | 1 | 1 | 1 | 1 | 1 | 1 | 1 | 1 | 1 | 1 |
| SMHenan9 | 1 | 1 | 1 | 1 | 1 | 1 | 1 | 1 | 1 | 1 | 1 | 1 | 1 | 1 | 1 | 1 | 1 | 1 | 1 | 1 | 1 |
| SMHenan10 | 1 | 1 | 1 | 1 | 1 | 1 | 1 | 1 | 1 | 1 | 1 | 1 | 1 | 1 | 1 | 1 | 1 | 1 | 1 | 1 | 1 |
| SMHenan11 | 1 | 1 | 1 | 1 | 1 | 1 | 1 | 1 | 1 | 1 | 1 | 1 | 1 | 1 | 1 | 1 | 1 | 1 | 1 | 1 | 1 |
| SMHenan12 | 1 | 1 | 1 | 1 | 1 | 1 | 1 | 1 | 1 | 1 | 1 | 1 | 1 | 1 | 1 | 1 | 1 | 1 | 1 | 1 | 1 |
| SMHenan13 | 1 | 1 | 1 | 1 | 1 | 1 | 1 | 1 | 1 | 1 | 1 | 1 | 1 | 1 | 1 | 1 | 1 | 1 | 1 | 1 | 1 |

| **Strain** | **Secretion system** | | | | | | | | | | | | | | | | | | | | | | | | | |
| --- | --- | --- | --- | --- | --- | --- | --- | --- | --- | --- | --- | --- | --- | --- | --- | --- | --- | --- | --- | --- | --- | --- | --- | --- | --- | --- |
|  | **TTSS (SPI-2 encode)** | | | | | | | | | | | | | | | | | | | | | | | | | |
|  | ***ssaC*** | ***ssaD*** | ***ssaE*** | ***ssaG*** | ***ssaH*** | ***ssaI*** | ***ssaJ*** | ***ssaK*** | ***ssaL*** | ***ssaM*** | ***ssaN*** | ***ssaO*** | ***ssaP*** | ***ssaQ*** | ***ssaR*** | ***ssaT*** | ***ssaU*** | ***ssaV*** | ***sscA*** | ***sscB*** | ***sseB*** | ***sseC*** | ***sseD*** | ***sseE*** | ***ssrA*** | ***ssrB*** |
| SMHunan1 | 1 | 1 | 1 | 1 | 1 | 0 | 1 | 1 | 1 | 1 | 1 | 1 | 1 | 1 | 1 | 1 | 1 | 1 | 1 | 1 | 1 | 1 | 1 | 1 | 1 | 1 |
| SMHunan2 | 1 | 1 | 1 | 1 | 1 | 1 | 1 | 1 | 1 | 1 | 1 | 1 | 1 | 1 | 1 | 1 | 1 | 1 | 1 | 1 | 1 | 1 | 1 | 1 | 1 | 1 |
| SMHunan3 | 1 | 1 | 1 | 1 | 1 | 1 | 1 | 1 | 1 | 1 | 1 | 1 | 1 | 1 | 1 | 1 | 1 | 1 | 1 | 1 | 1 | 1 | 1 | 1 | 1 | 1 |
| SMHunan4 | 1 | 1 | 1 | 1 | 1 | 0 | 1 | 1 | 1 | 1 | 1 | 1 | 1 | 1 | 1 | 1 | 1 | 1 | 1 | 1 | 1 | 1 | 1 | 1 | 1 | 1 |
| SMHunan5 | 1 | 1 | 1 | 1 | 1 | 1 | 1 | 1 | 1 | 1 | 1 | 1 | 1 | 1 | 1 | 1 | 1 | 1 | 1 | 1 | 1 | 1 | 1 | 1 | 1 | 1 |
| SMHunan6 | 1 | 1 | 1 | 1 | 1 | 1 | 1 | 1 | 1 | 1 | 1 | 1 | 1 | 1 | 1 | 1 | 1 | 1 | 1 | 1 | 1 | 1 | 1 | 1 | 1 | 1 |
| SMHenan1 | 1 | 1 | 1 | 1 | 1 | 0 | 1 | 1 | 1 | 1 | 1 | 1 | 1 | 1 | 1 | 1 | 1 | 1 | 1 | 1 | 1 | 1 | 1 | 1 | 1 | 1 |
| SMHenan2 | 1 | 1 | 1 | 1 | 1 | 0 | 1 | 1 | 1 | 1 | 1 | 1 | 1 | 1 | 1 | 1 | 1 | 1 | 1 | 1 | 1 | 1 | 1 | 1 | 1 | 1 |
| SMHenan3 | 1 | 1 | 1 | 1 | 1 | 0 | 1 | 1 | 1 | 1 | 1 | 1 | 1 | 1 | 1 | 1 | 1 | 1 | 1 | 1 | 1 | 1 | 1 | 1 | 1 | 1 |
| SMHenan4 | 1 | 1 | 1 | 1 | 1 | 0 | 1 | 1 | 1 | 1 | 1 | 1 | 1 | 1 | 1 | 1 | 1 | 1 | 1 | 1 | 1 | 1 | 1 | 1 | 1 | 1 |
| SMHenan5 | 1 | 1 | 1 | 1 | 1 | 0 | 1 | 1 | 1 | 1 | 1 | 1 | 1 | 1 | 1 | 1 | 1 | 1 | 1 | 1 | 1 | 1 | 1 | 1 | 1 | 1 |
| SMHebei1 | 1 | 1 | 1 | 1 | 1 | 1 | 1 | 1 | 1 | 1 | 1 | 1 | 1 | 1 | 1 | 1 | 1 | 1 | 1 | 1 | 1 | 1 | 1 | 1 | 1 | 1 |
| SMHebei2 | 1 | 1 | 1 | 1 | 1 | 0 | 1 | 1 | 1 | 1 | 1 | 1 | 1 | 1 | 1 | 1 | 1 | 1 | 1 | 1 | 1 | 1 | 1 | 1 | 1 | 1 |
| SMHebei3 | 1 | 1 | 1 | 1 | 1 | 0 | 1 | 1 | 1 | 1 | 1 | 1 | 1 | 1 | 1 | 1 | 1 | 1 | 1 | 1 | 1 | 1 | 1 | 1 | 1 | 1 |
| SMHebei4 | 1 | 1 | 1 | 1 | 1 | 1 | 1 | 1 | 1 | 1 | 1 | 1 | 1 | 1 | 1 | 1 | 1 | 1 | 1 | 1 | 1 | 1 | 1 | 1 | 1 | 1 |
| SMHebei5 | 1 | 1 | 1 | 1 | 1 | 1 | 1 | 1 | 1 | 1 | 1 | 1 | 1 | 1 | 1 | 1 | 1 | 1 | 1 | 1 | 1 | 1 | 1 | 1 | 1 | 1 |
| SMHebei6 | 1 | 1 | 1 | 1 | 1 | 0 | 1 | 1 | 1 | 1 | 1 | 1 | 1 | 1 | 1 | 1 | 1 | 1 | 1 | 1 | 1 | 1 | 1 | 1 | 1 | 1 |
| SMHebei7 | 1 | 1 | 1 | 1 | 1 | 1 | 1 | 1 | 1 | 1 | 1 | 1 | 1 | 1 | 1 | 1 | 1 | 1 | 1 | 1 | 1 | 1 | 1 | 1 | 1 | 1 |
| SMHebei8 | 1 | 1 | 1 | 1 | 1 | 0 | 1 | 1 | 1 | 1 | 1 | 1 | 1 | 1 | 1 | 1 | 1 | 1 | 1 | 1 | 1 | 1 | 1 | 1 | 1 | 1 |
| SMHebei9 | 1 | 1 | 1 | 1 | 1 | 1 | 1 | 1 | 1 | 1 | 1 | 1 | 1 | 1 | 1 | 1 | 1 | 1 | 1 | 1 | 1 | 1 | 1 | 1 | 1 | 1 |
| SMHebei10 | 1 | 1 | 1 | 1 | 1 | 0 | 1 | 1 | 1 | 1 | 1 | 1 | 1 | 1 | 1 | 1 | 1 | 1 | 1 | 1 | 1 | 1 | 1 | 1 | 1 | 1 |
| SMShanghai1 | 1 | 1 | 1 | 1 | 1 | 0 | 1 | 1 | 1 | 0 | 1 | 1 | 1 | 1 | 1 | 1 | 1 | 1 | 1 | 1 | 1 | 1 | 1 | 1 | 1 | 1 |
| SMShanghai2 | 1 | 1 | 1 | 1 | 1 | 1 | 1 | 1 | 1 | 1 | 1 | 1 | 1 | 1 | 1 | 1 | 1 | 1 | 1 | 1 | 1 | 1 | 1 | 1 | 1 | 1 |
| SMShanghai3 | 1 | 1 | 1 | 1 | 1 | 0 | 1 | 1 | 1 | 0 | 1 | 1 | 1 | 1 | 1 | 1 | 1 | 1 | 1 | 1 | 1 | 1 | 1 | 1 | 1 | 1 |
| SMHenan6 | 1 | 1 | 1 | 1 | 1 | 0 | 1 | 1 | 1 | 1 | 1 | 1 | 1 | 1 | 1 | 1 | 1 | 1 | 1 | 1 | 1 | 1 | 1 | 1 | 1 | 1 |
| SMHenan7 | 1 | 1 | 1 | 1 | 1 | 1 | 1 | 1 | 1 | 1 | 1 | 1 | 1 | 1 | 1 | 1 | 1 | 1 | 1 | 1 | 1 | 1 | 1 | 1 | 1 | 1 |
| SMHenan8 | 1 | 1 | 1 | 1 | 1 | 0 | 1 | 1 | 1 | 1 | 1 | 1 | 1 | 1 | 1 | 1 | 1 | 1 | 1 | 1 | 1 | 1 | 1 | 1 | 1 | 1 |
| SMHenan9 | 1 | 1 | 1 | 1 | 1 | 0 | 1 | 1 | 1 | 1 | 1 | 1 | 1 | 1 | 1 | 1 | 1 | 1 | 1 | 1 | 1 | 1 | 1 | 1 | 1 | 1 |
| SMHenan10 | 1 | 1 | 1 | 1 | 1 | 0 | 1 | 1 | 1 | 1 | 1 | 1 | 1 | 1 | 1 | 1 | 1 | 1 | 1 | 1 | 1 | 1 | 1 | 1 | 1 | 1 |
| SMHenan11 | 1 | 1 | 1 | 1 | 1 | 0 | 1 | 1 | 1 | 1 | 1 | 1 | 1 | 1 | 1 | 1 | 1 | 1 | 1 | 1 | 1 | 1 | 1 | 1 | 1 | 1 |
| SMHenan12 | 1 | 1 | 1 | 1 | 1 | 0 | 1 | 1 | 1 | 1 | 1 | 1 | 1 | 1 | 1 | 1 | 1 | 1 | 1 | 1 | 1 | 1 | 1 | 1 | 1 | 1 |
| SMHenan13 | 1 | 1 | 1 | 1 | 1 | 0 | 1 | 1 | 1 | 1 | 1 | 1 | 1 | 1 | 1 | 1 | 1 | 1 | 1 | 1 | 1 | 1 | 1 | 1 | 1 | 1 |

| **Strain** | **Secretion system** | | | | | | | | | | | | | | | | | | | | |
| --- | --- | --- | --- | --- | --- | --- | --- | --- | --- | --- | --- | --- | --- | --- | --- | --- | --- | --- | --- | --- | --- |
|  | **TTSS effectors translocated via**  **both systems** | **TTSS-1 translocated effectors** | | | | | | | | | **TTSS-2 translocated effectors** | | | | | | | | | | |
|  | ***slrP*** | ***avrA*** | ***sipA*** | ***sipB*** | ***sipC*** | ***sopA*** | ***sopB/sigD*** | ***sopD*** | ***sopE2*** | ***sptP*** | ***gogB*** | ***pipB2*** | ***pipB*** | ***sifA*** | ***sifB*** | ***sseF*** | ***sseG*** | ***sseI/srfH*** | ***sseJ*** | ***sseK1*** | ***sseK2*** |
| SMHunan1 | 1 | 1 | 1 | 1 | 1 | 1 | 1 | 1 | 1 | 1 | 1 | 1 | 1 | 1 | 1 | 1 | 0 | 1 | 1 | 1 | 1 |
| SMHunan2 | 1 | 1 | 1 | 1 | 1 | 1 | 1 | 1 | 1 | 1 | 1 | 1 | 1 | 1 | 1 | 1 | 1 | 1 | 1 | 1 | 1 |
| SMHunan3 | 1 | 1 | 1 | 1 | 1 | 1 | 1 | 1 | 1 | 1 | 1 | 1 | 1 | 1 | 1 | 1 | 1 | 1 | 1 | 1 | 1 |
| SMHunan4 | 1 | 1 | 1 | 1 | 1 | 1 | 1 | 1 | 1 | 1 | 1 | 1 | 1 | 1 | 1 | 1 | 0 | 1 | 1 | 1 | 1 |
| SMHunan5 | 1 | 1 | 1 | 1 | 1 | 1 | 1 | 1 | 1 | 1 | 1 | 1 | 1 | 1 | 1 | 1 | 1 | 1 | 1 | 1 | 1 |
| SMHunan6 | 1 | 1 | 1 | 1 | 1 | 1 | 1 | 1 | 1 | 1 | 1 | 1 | 1 | 1 | 1 | 1 | 1 | 1 | 1 | 1 | 1 |
| SMHenan1 | 1 | 1 | 1 | 1 | 1 | 1 | 1 | 1 | 1 | 1 | 1 | 1 | 1 | 1 | 1 | 1 | 0 | 1 | 1 | 1 | 1 |
| SMHenan2 | 1 | 1 | 1 | 1 | 1 | 1 | 1 | 1 | 1 | 1 | 1 | 1 | 1 | 1 | 1 | 1 | 0 | 1 | 1 | 1 | 1 |
| SMHenan3 | 1 | 1 | 1 | 1 | 1 | 1 | 1 | 1 | 1 | 1 | 1 | 1 | 1 | 1 | 1 | 1 | 0 | 1 | 1 | 1 | 1 |
| SMHenan4 | 1 | 1 | 1 | 1 | 1 | 1 | 1 | 1 | 1 | 1 | 1 | 1 | 1 | 1 | 1 | 1 | 0 | 1 | 1 | 1 | 1 |
| SMHenan5 | 1 | 1 | 1 | 1 | 1 | 1 | 1 | 1 | 1 | 1 | 1 | 1 | 1 | 1 | 1 | 1 | 0 | 1 | 1 | 1 | 1 |
| SMHebei1 | 1 | 1 | 1 | 1 | 1 | 1 | 1 | 1 | 1 | 1 | 1 | 1 | 1 | 1 | 1 | 1 | 1 | 1 | 1 | 1 | 1 |
| SMHebei2 | 1 | 1 | 1 | 1 | 1 | 1 | 1 | 1 | 1 | 1 | 1 | 1 | 1 | 1 | 1 | 1 | 0 | 1 | 1 | 1 | 1 |
| SMHebei3 | 1 | 1 | 1 | 1 | 1 | 1 | 1 | 1 | 1 | 1 | 1 | 1 | 1 | 1 | 1 | 1 | 0 | 1 | 1 | 1 | 1 |
| SMHebei4 | 1 | 1 | 1 | 1 | 1 | 1 | 1 | 1 | 1 | 1 | 1 | 1 | 1 | 1 | 1 | 1 | 1 | 1 | 1 | 1 | 1 |
| SMHebei5 | 1 | 1 | 1 | 1 | 1 | 1 | 1 | 1 | 1 | 1 | 1 | 1 | 1 | 1 | 1 | 1 | 1 | 1 | 1 | 1 | 1 |
| SMHebei6 | 1 | 1 | 1 | 1 | 1 | 1 | 1 | 1 | 1 | 1 | 1 | 1 | 1 | 1 | 1 | 1 | 0 | 1 | 1 | 1 | 1 |
| SMHebei7 | 1 | 1 | 1 | 1 | 1 | 1 | 1 | 1 | 1 | 1 | 1 | 1 | 1 | 1 | 1 | 1 | 0 | 1 | 1 | 1 | 1 |
| SMHebei8 | 1 | 1 | 1 | 1 | 1 | 1 | 1 | 1 | 1 | 1 | 1 | 1 | 1 | 1 | 1 | 1 | 0 | 1 | 1 | 1 | 1 |
| SMHebei9 | 1 | 1 | 1 | 1 | 1 | 1 | 1 | 1 | 1 | 1 | 1 | 1 | 1 | 1 | 1 | 1 | 1 | 1 | 1 | 1 | 1 |
| SMHebei10 | 1 | 1 | 1 | 1 | 1 | 1 | 1 | 1 | 1 | 1 | 1 | 1 | 1 | 1 | 1 | 1 | 0 | 1 | 1 | 1 | 1 |
| SMShanghai1 | 1 | 1 | 1 | 1 | 1 | 1 | 1 | 1 | 1 | 1 | 1 | 1 | 1 | 1 | 1 | 1 | 0 | 1 | 1 | 1 | 1 |
| SMShanghai2 | 1 | 1 | 1 | 1 | 1 | 1 | 1 | 1 | 1 | 1 | 1 | 1 | 1 | 1 | 1 | 1 | 1 | 1 | 1 | 1 | 1 |
| SMShanghai3 | 1 | 1 | 1 | 1 | 1 | 1 | 1 | 1 | 1 | 1 | 1 | 1 | 1 | 1 | 1 | 1 | 0 | 1 | 1 | 1 | 1 |
| SMHenan6 | 1 | 1 | 1 | 1 | 1 | 1 | 1 | 1 | 1 | 1 | 1 | 1 | 1 | 1 | 1 | 1 | 0 | 1 | 1 | 1 | 1 |
| SMHenan7 | 1 | 1 | 1 | 1 | 1 | 1 | 1 | 1 | 1 | 1 | 1 | 1 | 1 | 1 | 1 | 1 | 1 | 1 | 1 | 1 | 1 |
| SMHenan8 | 1 | 1 | 1 | 1 | 1 | 1 | 1 | 1 | 1 | 1 | 1 | 1 | 1 | 1 | 1 | 1 | 0 | 1 | 1 | 1 | 1 |
| SMHenan9 | 1 | 1 | 1 | 1 | 1 | 1 | 1 | 1 | 1 | 1 | 1 | 1 | 1 | 1 | 1 | 1 | 0 | 1 | 1 | 1 | 1 |
| SMHenan10 | 1 | 1 | 1 | 1 | 1 | 1 | 1 | 1 | 1 | 1 | 1 | 1 | 1 | 1 | 1 | 1 | 0 | 1 | 1 | 1 | 1 |
| SMHenan11 | 1 | 1 | 1 | 1 | 1 | 1 | 1 | 1 | 1 | 1 | 1 | 1 | 1 | 1 | 1 | 1 | 0 | 1 | 1 | 1 | 1 |
| SMHenan12 | 1 | 1 | 1 | 1 | 1 | 1 | 1 | 1 | 1 | 1 | 1 | 1 | 1 | 1 | 1 | 1 | 0 | 1 | 1 | 1 | 1 |
| SMHenan13 | 1 | 1 | 1 | 1 | 1 | 1 | 1 | 1 | 1 | 1 | 1 | 1 | 1 | 1 | 1 | 1 | 0 | 1 | 1 | 1 | 1 |

| **Strain** | **Secretion system** | | **Serum resistance** | **Stress adaptation** | **Toxin** |
| --- | --- | --- | --- | --- | --- |
|  | **TTSS-2 translocated effectors** | | **Rck** | **SodCI** | **SpvB** |
|  | ***sseL*** | ***sspH2*** | ***rck*** | ***sodCI*** | ***spvB*** |
| SMHunan1 | 1 | 1 | 1 | 1 | 1 |
| SMHunan2 | 1 | 1 | 1 | 1 | 1 |
| SMHunan3 | 1 | 1 | 1 | 1 | 1 |
| SMHunan4 | 1 | 1 | 1 | 1 | 1 |
| SMHunan5 | 1 | 1 | 1 | 1 | 1 |
| SMHunan6 | 1 | 1 | 1 | 1 | 1 |
| SMHenan1 | 1 | 1 | 1 | 1 | 1 |
| SMHenan2 | 1 | 1 | 1 | 1 | 1 |
| SMHenan3 | 1 | 1 | 1 | 1 | 1 |
| SMHenan4 | 1 | 1 | 1 | 1 | 1 |
| SMHenan5 | 1 | 1 | 1 | 1 | 1 |
| SMHebei1 | 1 | 1 | 1 | 1 | 1 |
| SMHebei2 | 1 | 1 | 1 | 1 | 1 |
| SMHebei3 | 1 | 1 | 1 | 1 | 1 |
| SMHebei4 | 1 | 1 | 1 | 1 | 1 |
| SMHebei5 | 1 | 1 | 1 | 1 | 1 |
| SMHebei6 | 1 | 1 | 1 | 1 | 1 |
| SMHebei7 | 1 | 1 | 1 | 1 | 1 |
| SMHebei8 | 1 | 0 | 1 | 1 | 1 |
| SMHebei9 | 1 | 1 | 1 | 1 | 1 |
| SMHebei10 | 1 | 1 | 1 | 1 | 1 |
| SMShanghai1 | 1 | 1 | 1 | 1 | 1 |
| SMShanghai2 | 1 | 1 | 1 | 1 | 1 |
| SMShanghai3 | 1 | 1 | 1 | 1 | 1 |
| SMHenan6 | 1 | 1 | 1 | 1 | 1 |
| SMHenan7 | 1 | 1 | 1 | 1 | 1 |
| SMHenan8 | 1 | 1 | 1 | 1 | 1 |
| SMHenan9 | 1 | 1 | 1 | 1 | 1 |
| SMHenan10 | 1 | 1 | 1 | 1 | 1 |
| SMHenan11 | 1 | 1 | 1 | 1 | 1 |
| SMHenan12 | 1 | 1 | 1 | 1 | 1 |
| SMHenan13 | 1 | 1 | 1 | 1 | 1 |

| **Strain** | **Phenotypic AMR** | | | | | | | | | | | | |
| --- | --- | --- | --- | --- | --- | --- | --- | --- | --- | --- | --- | --- | --- |
|  | **Penicillins** | | | | | **Carbapenems** | | | | **Cephalosporins** | | | |
|  | **Ampicillin** | **Piperacillin** | **Ticarcillin/Clavulanic Acid** | **Ampicillin/Sulbactam** | **Piperacillin/Tazobactam** | **Meropenem** | **Eertapenem** | **Imipenem** | **Doripenem** | **Cefazolin** | **Cefepime** | **Ceftazidime** | **Ceftriaxone** |
| SMHunan1 | <8 | <16 | <8/2 | <8/4 | <16/4 | <0.5 | <0.25 | <0.5 | <0.5 | >8 | <4 | <1 | <0.5 |
| SMHunan2 | <8 | 32-64 | <8/2 | <8/4 | 32/4 | <0.5 | <0.25 | <0.5 | <0.5 | >8 | <4 | <1 | <0.5 |
| SMHunan3 | <8 | <16 | <8/2 | <8/4 | <16/4 | <0.5 | <0.25 | <0.5 | <0.5 | >8 | <4 | <1 | <0.5 |
| SMHunan4 | <8 | <16 | 32/2-64/2 | <8/4 | <16/4 | <0.5 | <0.25 | <0.5 | <0.5 | >8 | <4 | <1 | <0.5 |
| SMHunan5 | <8 | <16 | <8/2 | <8/4 | <16/4 | <0.5 | <0.25 | <0.5 | <0.5 | >8 | <4 | <1 | <0.5 |
| SMHunan6 | <8 | <16 | <8/2 | <8/4 | <16/4 | <0.5 | <0.25 | <0.5 | <0.5 | >8 | <4 | <1 | <0.5 |
| SMHenan1 | <8 | <16 | <8/2 | <8/4 | <16/4 | <0.5 | <0.25 | <0.5 | <0.5 | >8 | <4 | <1 | <0.5 |
| SMHenan2 | <8 | <16 | <8/2 | <8/4 | <16/4 | <0.5 | <0.25 | <0.5 | <0.5 | >8 | <4 | <1 | <0.5 |
| SMHenan3 | 16 | <16 | <8/2 | <8/4 | <16/4 | <0.5 | <0.25 | <0.5 | <0.5 | >8 | <4 | <1 | <0.5 |
| SMHenan4 | <8 | <16 | <8/2 | <8/4 | <16/4 | <0.5 | <0.25 | <0.5 | <0.5 | >8 | <4 | <1 | <0.5 |
| SMHenan5 | <8 | <16 | <8/2 | <8/4 | <16/4 | <0.5 | <0.25 | 2 | <0.5 | >8 | <4 | <1 | <0.5 |
| SMHebei1 | <8 | <16 | 32/2-64/2 | <8/4 | <16/4 | <0.5 | <0.25 | <0.5 | <0.5 | 16 | <4 | 8 | <0.5 |
| SMHebei2 | <8 | <16 | <8/2 | <8/4 | <16/4 | <0.5 | <0.25 | <0.5 | <0.5 | >8 | <4 | <1 | <0.5 |
| SMHebei3 | <8 | <16 | <8/2 | <8/4 | <16/4 | <0.5 | <0.25 | <0.5 | <0.5 | >8 | <4 | <1 | <0.5 |
| SMHebei4 | 16 | 32-64 | <8/2 | 16/8 | <16/4 | 2 | <0.25 | <0.5 | <0.5 | >8 | <4 | <1 | <0.5 |
| SMHebei5 | <8 | <16 | <8/2 | <8/4 | <16/4 | <0.5 | <0.25 | <0.5 | <0.5 | >8 | <4 | <1 | <0.5 |
| SMHebei6 | <8 | <16 | <8/2 | <8/4 | <16/4 | <0.5 | <0.25 | <0.5 | <0.5 | >8 | <4 | <1 | <0.5 |
| SMHebei7 | <8 | <16 | <8/2 | <8/4 | <16/4 | <0.5 | <0.25 | <0.5 | <0.5 | >8 | <4 | <1 | <0.5 |
| SMHebei8 | <8 | <16 | <8/2 | <8/4 | <16/4 | <0.5 | <0.25 | <0.5 | <0.5 | >8 | <4 | <1 | <0.5 |
| SMHebei9 | <8 | <16 | <8/2 | <8/4 | <16/4 | <0.5 | <0.25 | <0.5 | <0.5 | >8 | <4 | <1 | <0.5 |
| SMHebei10 | <8 | <16 | <8/2 | <8/4 | <16/4 | <0.5 | <0.25 | <0.5 | <0.5 | >8 | <4 | <1 | <0.5 |
| SMShanghai1 | <8 | <16 | <8/2 | <8/4 | <16/4 | <0.5 | <0.25 | <0.5 | <0.5 | >8 | <4 | <1 | <0.5 |
| SMShanghai2 | <8 | <16 | <8/2 | <8/4 | >128/4 | <0.5 | <0.25 | <0.5 | <0.5 | >8 | <4 | <1 | <0.5 |
| SMShanghai3 | <8 | <16 | <8/2 | <8/4 | <16/4 | <0.5 | <0.25 | <0.5 | <0.5 | >8 | <4 | <1 | <0.5 |
| SMHenan6 | <8 | <16 | <8/2 | <8/4 | 32/4 | <0.5 | <0.25 | <0.5 | <0.5 | >8 | <4 | <1 | <0.5 |
| SMHenan7 | <8 | 32-64 | <8/2 | <8/4 | <16/4 | <0.5 | <0.25 | <0.5 | <0.5 | >8 | <4 | <1 | <0.5 |
| SMHenan8 | <8 | <16 | <8/2 | <8/4 | <16/4 | <0.5 | <0.25 | <0.5 | <0.5 | >8 | <4 | <1 | <0.5 |
| SMHenan9 | <8 | <16 | <8/2 | <8/4 | <16/4 | <0.5 | <0.25 | <0.5 | <0.5 | >8 | <4 | <1 | <0.5 |
| SMHenan10 | <8 | 32-64 | <8/2 | <8/4 | <16/4 | <0.5 | <0.25 | <0.5 | <0.5 | >8 | <4 | <1 | <0.5 |
| SMHenan11 | <8 | 32-64 | <8/2 | <8/4 | >128/4 | <0.5 | <0.25 | <0.5 | <0.5 | >8 | <4 | <1 | <0.5 |
| SMHenan12 | <8 | 32-64 | <8/2 | <8/4 | >128/4 | <0.5 | <0.25 | <0.5 | <0.5 | >8 | <4 | <1 | <0.5 |
| SMHenan13 | <8 | 32-64 | <8/2 | <8/4 | <16/4 | <0.5 | <0.25 | <0.5 | <0.5 | >8 | <4 | <1 | <0.5 |

| **Strain** | **Phenotypic AMR** | | | | | | | | | | |
| --- | --- | --- | --- | --- | --- | --- | --- | --- | --- | --- | --- |
|  | **Monobactams** | **Aminoglycosides** | | | **Tetracyclines** | | | **Quinolones** | | **Folate Pathway Inhibitors** | **Nitrofuran** |
|  | **Aztreonam** | **Gentamicin** | **Amikacin** | **Tobramycin** | **Tetracycline** | **Minocycline** | **Tigecycline** | **Ciprofloxacin** | **Levofloxacin** | **Trimethoprim/Sulfamethoxazole** | **Nitrofurantoin** |
| SMHunan1 | <1 | >8 | >64 | >16 | 8 | 8 | 8 | <0.06 | <1 | >4/76 | <32 |
| SMHunan2 | <1 | >8 | >64 | >16 | 8 | 8 | <1 | <0.06 | <1 | <2/38 | 64 |
| SMHunan3 | <1 | >8 | >64 | >16 | 8 | 8 | 8 | <0.06 | <1 | <2/38 | <32 |
| SMHunan4 | <1 | >8 | >64 | >16 | 8 | 8 | 4 | <0.06 | <1 | <2/38 | <32 |
| SMHunan5 | <1 | >8 | >64 | >16 | <4 | <1 | <1 | <0.06 | <1 | <2/38 | <32 |
| SMHunan6 | <1 | >8 | >64 | >16 | <4 | <1 | <1 | <0.06 | <1 | <2/38 | <32 |
| SMHenan1 | <1 | >8 | >64 | >16 | 8 | 8 | 4 | <0.06 | <1 | <2/38 | <32 |
| SMHenan2 | <1 | >8 | >64 | >16 | 8 | 8 | 4 | <0.06 | <1 | <2/38 | <32 |
| SMHenan3 | <1 | >8 | >64 | >16 | 8 | 8 | 4 | <0.06 | <1 | <2/38 | <32 |
| SMHenan4 | <1 | >8 | >64 | >16 | <4 | 8 | 4 | <0.06 | <1 | <2/38 | <32 |
| SMHenan5 | <1 | >8 | >64 | >16 | 8 | 8 | <1 | <0.06 | <1 | <2/38 | <32 |
| SMHebei1 | <1 | >8 | >64 | >16 | 8 | 8 | 8 | <0.06 | <1 | >4/76 | <32 |
| SMHebei2 | <1 | >8 | >64 | >16 | 8 | 8 | 4 | <0.06 | <1 | <2/38 | <32 |
| SMHebei3 | <1 | >8 | >64 | >16 | >8 | 8 | 4 | <0.06 | <1 | <2/38 | <32 |
| SMHebei4 | <1 | >8 | >64 | >16 | 8 | 8 | 4 | >1 | <1 | >4/76 | <32 |
| SMHebei5 | <1 | >8 | >64 | >16 | 8 | 8 | <1 | <0.06 | <1 | <2/38 | <32 |
| SMHebei6 | <1 | >8 | >64 | >16 | 8 | 8 | 4 | 0.12-0.5 | <1 | <2/38 | <32 |
| SMHebei7 | <1 | >8 | >64 | >16 | 8 | 8 | <1 | 0.12-0.5 | <1 | <2/38 | <32 |
| SMHebei8 | <1 | >8 | >64 | >16 | 8 | 8 | <1 | 0.12-0.5 | <1 | <2/38 | <32 |
| SMHebei9 | <1 | >8 | >64 | >16 | 8 | 8 | <1 | 0.12-0.5 | <1 | <2/38 | <32 |
| SMHebei10 | <1 | >8 | >64 | >16 | <4 | <1 | <1 | <0.06 | <1 | <2/38 | <32 |
| SMShanghai1 | <1 | >8 | >64 | >16 | <4 | 8 | <1 | <0.06 | <1 | <2/38 | <32 |
| SMShanghai2 | <1 | >8 | >64 | >16 | 8 | 8 | <1 | <0.06 | <1 | <2/38 | <32 |
| SMShanghai3 | <1 | >8 | >64 | >16 | 8 | 8 | <1 | <0.06 | <1 | <2/38 | <32 |
| SMHenan6 | <1 | >8 | >64 | >16 | <4 | <1 | <1 | <0.06 | <1 | <2/38 | <32 |
| SMHenan7 | <1 | >8 | >64 | >16 | 8 | 8 | 4 | <0.06 | <1 | <2/38 | <32 |
| SMHenan8 | <1 | >8 | >64 | >16 | 8 | 8 | <1 | <0.06 | <1 | <2/38 | <32 |
| SMHenan9 | <1 | >8 | >64 | >16 | 8 | 8 | <1 | <0.06 | <1 | <2/38 | <32 |
| SMHenan10 | <1 | >8 | >64 | >16 | 8 | 8 | 4 | <0.06 | <1 | <2/38 | <32 |
| SMHenan11 | <1 | >8 | >64 | >16 | <4 | <1 | 4 | <0.06 | <1 | <2/38 | <32 |
| SMHenan12 | <1 | >8 | >64 | >16 | 8 | 8 | <1 | <0.06 | <1 | <2/38 | 64 |
| SMHenan13 | <1 | >8 | >64 | >16 | 8 | 8 | 4 | <0.06 | <1 | <2/38 | <32 |
